# Signaling Networks Converge on TORC1-SREBP Activity to Promote Endoplasmic Reticulum Homeostasis

**DOI:** 10.1371/journal.pone.0101164

**Published:** 2014-07-09

**Authors:** Miguel Sanchez-Alvarez, Fabian Finger, Maria del Mar Arias-Garcia, Vicky Bousgouni, Patricia Pascual-Vargas, Chris Bakal

**Affiliations:** Division of Cancer Biology, Chester Beatty Laboratories, Institute of Cancer Research, London, United Kingdom; University of Hong Kong, Hong Kong

## Abstract

The function and capacity of the endoplasmic reticulum (ER) is determined by multiple processes ranging from the local regulation of peptide translation, translocation, and folding, to global changes in lipid composition. ER homeostasis thus requires complex interactions amongst numerous cellular components. However, describing the networks that maintain ER function during changes in cell behavior and environmental fluctuations has, to date, proven difficult. Here we perform a systems-level analysis of ER homeostasis, and find that although signaling networks that regulate ER function have a largely modular architecture, the TORC1-SREBP signaling axis is a central node that integrates signals emanating from different sub-networks. TORC1-SREBP promotes ER homeostasis by regulating phospholipid biosynthesis and driving changes in ER morphology. In particular, our network model shows TORC1-SREBP serves to integrate signals promoting growth and G1-S progression in order to maintain ER function during cell proliferation.

## Introduction

The endoplasmic reticulum (ER) harbors the cellular machinery responsible for protein folding, maturation, trafficking and secretion; calcium homeostasis; and the metabolism of complex lipids. ER homeostasis must be maintained during cellular events that place a functional demand on the ER, such as cell growth and proliferation, differentiation, or activation of secretion, as well as during fluctuations in environmental conditions. Imbalances between the existing demand on the ER and its throughput are generically termed “ER stress”. ER stress can occur following accumulation of misfolded proteins, viral infection, imbalance in the composition of dietary lipids, disruption of ER calcium stores, and prolonged starvation [1]. An ancient signaling system, termed the Unfolded Protein Response (UPR), has evolved to continuously monitor the luminal ER environment, and engage a cellular program that promotes ER homeostasis [2]–[4]. The UPR comprises at least three sub-branches in higher eukaryotes, each dependent on a specific molecular transducer: Inositol Requiring Enzyme 1 (IRE1), PKR-like Endoplasmic Reticulum associated Kinase (PERK) and Activating Transcription Factor 6 (ATF6). PERK is a serine/threonine kinase that phosphorylates, upon activation, the eukaryotic translation initiation factor 2A (eIF2α), thus mainly attenuating protein synthesis. ATF6 is cleaved in ER stress conditions to yield a transcription activator controlling distinct adaptive programs. The most conserved branch relies on IRE1, a transmembrane kinase and RNAse that is localized to the ER membrane. Upon engagement of ER stress, IRE1 catalyzes the splicing of a short fragment of the mRNA encoded by the X-box Binding Protein 1 (*XBP1)* gene, eliciting its full translation as a potent transcriptional transactivator. The UPR can engage additional mechanisms, such as endoplasmic reticulum associated degradation (ERAD); reduction of anterograde vesicle transport to Golgi and increase in retrograde, Coat Protein complex I (COPI)-dependent transport; facilitation of autophagy activation; or even apoptosis in cases of chronic and/or severe ER stress [2]–[9].

The size, composition and architecture of ER membranes are clearly important factors in maintaining ER homeostasis. For example, UPR-mediated ER expansion during ER stress promotes homeostasis by increasing the luminal volume, which is thought to decrease the effective concentration of unfolded peptides and the probability of proteotoxic aggregation [10]. ER expansion is directly dependent on the activation of transcriptional programs driving phospholipid synthesis and mobilization [10]–[12]. In addition to ER volume, the relative composition of the ER membrane (for example, the ratio between phosphatidylcholine (PC) and phosphatidylethanolamine (PE), its two major phospholipid constituents) is also critical to ER homeostasis, as disruption of the PC:PE ratio leads to ER stress, aberrant calcium homeostasis, and may contribute to the pathogenesis of metabolic disease [13], [14].

The UPR is often activated in the absence of exogenous stress, suggesting that ER function must be coordinated with normal growth and proliferation. Basal IRE1 and/or XBP1 activity has been observed in proliferating yeast cells, and in diverse cell types and tissues such as B- and T-cells, dendritic cells, and in the placenta [1], [15]–[19]. In B-cells and thyrocytes, the UPR is transiently activated by growth stimuli and required for differentiation and activation, promoting ER expansion as part of a proactive measure to accommodate subsequent increases in secretory activity [19]. How signaling networks coordinate the maintenance of ER function, and the engagement of the UPR, with other cellular processes such as cell growth, proliferation, survival, and differentiation is poorly understood. Potentially, different signaling pathways or complexes could independently regulate different aspects of ER function, or alternatively there may exist one central gatekeeper, which integrates information from diverse sources to regulate ER homeostasis under physiological conditions. However, to date there has been no systems-level analysis of ER homeostasis that provides insight into the architecture and dynamics of the signaling networks that regulate ER function.

To gain insight into the signaling networks regulating ER function in metazoans, we have performed a series of quantitative genome-scale image-based analyses in *Drosophila* cells. These studies reveal that the network regulating ER homeostasis is largely modular in nature, and comprised of different sub-networks that also regulate diverse cellular processes in addition to ER function. For example, we show that signaling via the Target Of Rapamycin Complex 1 (TORC1) sub-network, a key regulator of positive growth signaling and anabolism is essential for ER homeostasis. Genetic experiments reveal that the vast majority of modules that regulate ER function are epistatic to the transcription factor Sterol Regulatory Element Binding Protein (SREBP), a regulator of lipid metabolism and a conserved target of TOR signaling. Furthermore, biochemical experiments show that exogenous lipids can rescue many defects in ER function induced by depletion of genes in the network, which shows that many sub-networks regulate ER homeostasis via SREBP-mediated control of lipid metabolism. Thus, we propose that TOR-SREBP mediated control of lipid metabolism serves as a central regulator of ER homeostasis, in part by integrating diverse signals emanating from different modular complexes, especially during growth and proliferation.

## Results

### Genome-scale screening identifies TOR as an essential regulator of IRE1 activity

To identify genes that contribute to ER homeostasis in proliferating cells, we adapted a previously developed image-based readout to monitor XBP1 splicing, and thus IRE1 RNAse activity, in *Drosophila* S2R+ cells [20]. Briefly, unconventional splicing of a highly conserved 23-nucleotide motif in the XBP1-EGFP (Enhanced Green Fluorescent Protein) mRNA results in EGFP-positive cells (fig. S1A and Methods). This reporter demonstrates dose-dependent sensitivity to ER stress induced by the protein glycosylation inhibitor tunicamycin (TM) (fig. S1B). Knockdown of IRE1 significantly decreases the percentage of EGFP positive cells following incubation with TM, demonstrating that its splicing activation is dependent on IRE1 RNAse activity (fig. S1B). We screened custom RNAi libraries for genes whose depletion increased or decreased the ratio of EGFP positive cells. The RNAi libraries included dsRNAs targeting all annotated *Drosophila* kinases and phosphatases (K/P set); the full repertoire of transcription factors and DNA binding proteins (TXN set); different well-established UPR regulators, protein chaperones, and RNA metabolism factors (ER/R set); and a subset of Rho GTPase Enhancing Factors and Activating Factors (RhoGEFs and RhoGAPs) in *Drosophila* (GEFGAP set) (Table S1 and fig. S1D).

**Figure 1:**
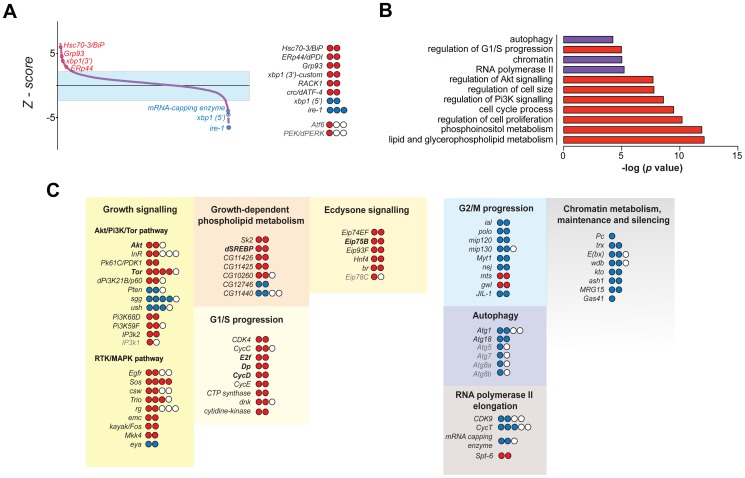
A genome-scale RNAi screen in S2R+ cells reveals regulators of ER homeostasis in normal proliferating cells. *(A)* Distribution of the ranked Z-scores for all interrogated genes. Expected hits, such as ER-resident chaperones, and UPR transducers are depicted. Color circles represent positive- (red), negative- (blue), or non-significant- (white), scoring amplicons for each specified gene. *(B)* GO functional enrichment analysis of the hit lists. *Red* categories predominantly comprehend positive-scoring genes; *blue* denote categories composed mainly by negative-scoring genes. *(C)* Major functional categories and components. Color circles represent positive- (red) negative- (blue) or non-significant- (white) scoring amplicons for each specified gene.

We consider a gene as a hit when depletion using two or more independent amplicons increases or decreases the percentage of EGFP positive cells above or below 1.5 standard deviations of the mean percentage of wild-type cells (fig. 1A; see Methods). As expected, depletion of IRE1 by RNAi consistently leads to the most significant decreases in XBP1-EGFP splicing (fig. 1A). Importantly, a dsRNA targeting specifically only the 3′-end of endogenous XBP1 (and therefore not the reporter construct) increases XBP1-EGFP levels, suggesting that XBP1 itself is required for ER homeostasis in proliferating S2R+ cells (see Table S1). Depletion of canonical ER-resident protein chaperones including Hsc70-3/BiP, Gp93/HSP90B1 and ERp44/PDIA3 increases ER stress (fig. 1A). In secondary screens in IRE1-deficient cells, we failed to identify significant hits (fig. S1C), further supporting the specificity of our readout.

Out of 237 primary hits, 186 were further validated by screening a manually curated sub-library, comprised of dsRNAs rederived from genomic DNA (fig. S1D; Table S1). Hits are significantly enriched in a number of GO terms such as “regulation of cell size”, “regulation of phosphoinositide 3-kinase (PI3K) signaling”, and “phospholipid metabolism” (fig. 1B). Based on their known function we grouped major hits into 4 classes (fig. 1C): i) PI3K-AKT-TOR signaling components including Insulin-like Receptor (InR), Protein kinase B (PKB/AKT), Phosphoinositide dependent kinase 1 (Pk61C/PDK1), and TOR; and Receptor Tyrosine Kinase (RTK)-Mitogen Activated Protein Kinase (MAPK) signaling components such as Epidermal Growth Factor receptor (Egfr), Trio and Son of Sevenless (Sos); ii) regulators of phospholipid metabolism, including the master transcriptional regulator SREBP; iii) promoters of cell cycle progression through G1/S; and iv) genes involved in transcriptional regulation of ecdysone signaling. Genes whose depletion suppressed baseline levels of ER stress include genes that contribute to: i) G2/M progression; ii) autophagy; iii) RNA polymerase II activity; and iv) chromatin architecture. Notably, depletion of genes that encode antagonists of PI3K signaling, such as Phosphatase Tensin homolog (PTEN) or Glycogen Synthase Kinase-3β (shaggy/GSK3β) also decreased the baseline levels of ER stress (fig. 1C). Importantly, while depletion of some genes such as AKT, TOR, and PTEN affected both XBP1-EGFP splicing and cell growth, we find no correlation between nuclear size (a proxy for cell size; [21] and XBP1-EGFP positive cells across the dataset (fig. S1E). This is best highlighted by the fact that deficiency of factors such as Dp or Cyclin D (CycD) leads to G1-arrested, large cells that exhibit XBP1 splicing scores comparable to smaller, TOR-deficient cells (fig. S1E). We conclude that growth and proliferation signaling, but not cell size *per se*, directly influence ER homeostasis in normal conditions. Taken together these results demonstrate that diverse signaling modules that regulate distinct processes such as growth, proliferation, autophagy, and chromatin architecture, also regulate ER function directly or indirectly.

### Sustained TORC1 Inhibition disrupts ER homeostasis

**Figure 2:**
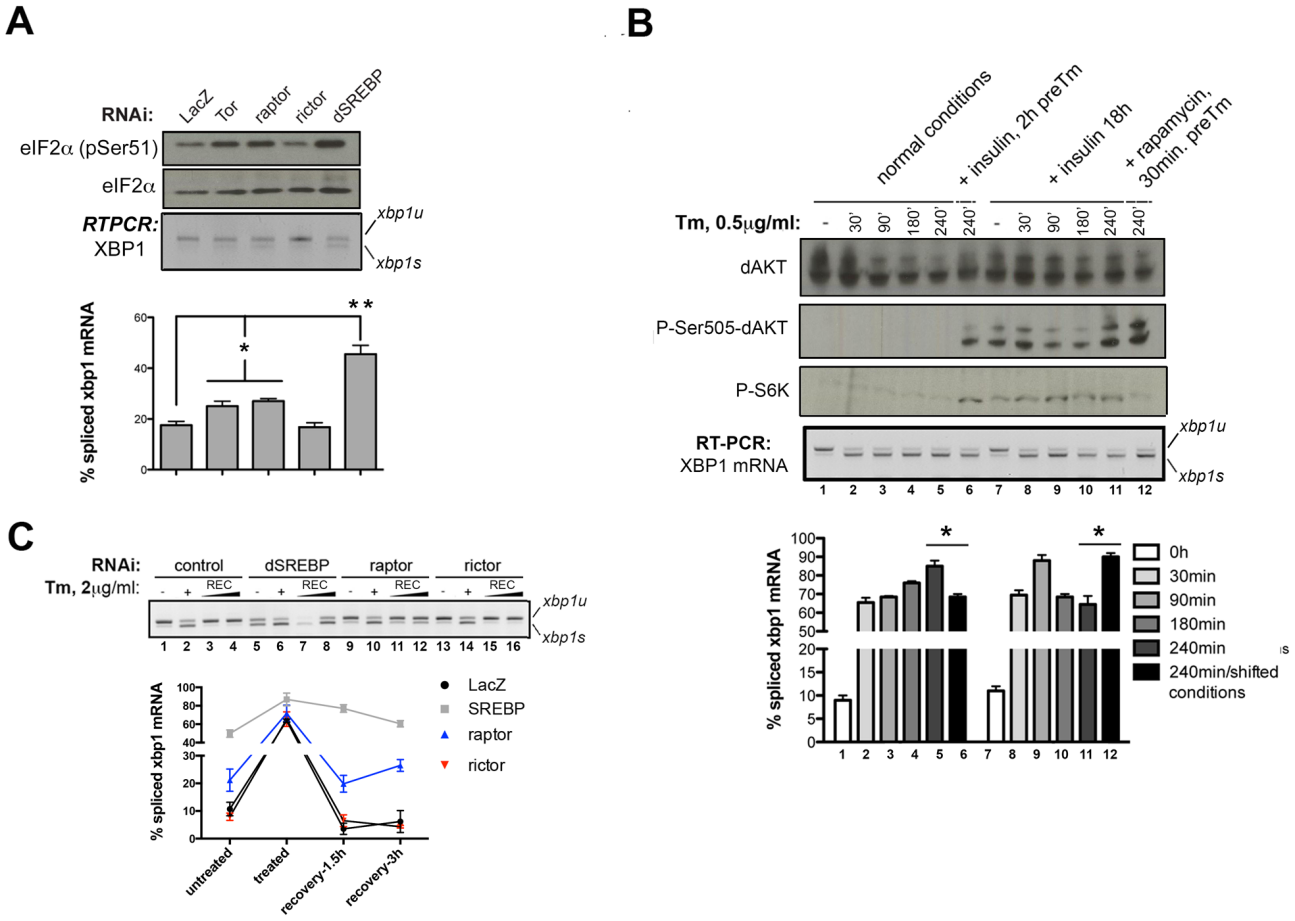
TORC1-dependent signaling is required for ER homeostasis and recovery from ER stress in *Drosophila* cells. *(A)* S2R+ cells transfected for 96 h with the indicated dsRNAs were processed for RT-PCR to detect endogenous *XBP1* forms, or western blot with the indicated antibodies. Graph shows the relative quantification of unspliced (*xbp1u*) and spliced (*xbp1s*) species for XBP1 mRNAs from two independent experiments performed in duplicate. *(B)* S2R+ cells were treated with TM for the indicated times. Preinduction with insulin (1 µM) was applied as indicated. Rapamycin treatment (20 nM) was applied as indicated. Total RNA was extracted and analyzed by RT-PCR for splicing forms of endogenous *XBP1* mRNA. Quantification graph shown was sourced from three independent experiments, each including two technical replicates. Western blot from analogous samples are shown for the analysis of total AKT, phospho-AKT and phospho-S6 relative levels. *(C)* S2R+ cells transfected for 96 h with the indicated dsRNAs were treated with vehicle (-) or TM (+), or pretreated with TM and then let recover for 3 or 5 h in fresh growth medium. Samples were then processed for RT-PCR to detect endogenous *XBP1* mRNA forms and perform relative quantitation as shown in the graph. Quantification shown was sourced from two independent experiments, each including two technical replicates. Where indicated, statistical analysis using t-Student's test was applied. *: *p*<0.05; **: *p*<0.01.

The increased levels of spliced XBP1-EGFP following TOR depletion is somewhat unexpected given that protein translation, and therefore the input of ER client proteins, are likely decreased in TOR-deficient cells [22]. Thus, we sought to further investigate the contribution of TOR signaling to ER homeostasis. We first monitored the increase in splicing of endogenous XBP1 by RT-PCR following RNAi depletion of TOR, as well as the positive hit SREBP (fig. 2A; see below). TOR is part of two distinct functional complexes, TORC1 and TORC2 [23]–[25]. In order to determine whether increases in ER stress following the depletion of TOR are due to defects in TORC1 and/or TORC2 signaling, we quantified XBP1 splicing following depletion of Raptor (a TORC1 specific component) or Rictor (a TORC2 specific component). Sustained depletion of TOR, Raptor and SREBP, but not Rictor, significantly increases the levels of XBP1 splicing in proliferating cells (fig. 2A). Furthermore, Western blot analysis revealed that eIF2α phosphorylation levels are increased in TOR, Raptor, and SREBP deficient cells, but not in Rictor deficient cells, suggesting that loss of TORC1 signaling leads to PERK activation (fig. 2A). These observations suggest that inhibiting TORC1 or its downstream target SREBP activates the UPR in metazoan cells.

We next sought to determine if activation of TOR signaling by insulin could influence the dynamics of the response to ER stress. Insulin stimulation for 18 h prior to exposure to TM favored a quicker attenuation of XBP1 splicing after 4 h of ER stress induction (fig. 2B; lanes 7–11 as compared with lanes 2–5). A short stimulation of 2 h before the start of the stressing stimulus also favored attenuation after TM exposure as compared with non-stimulated cells exposed for the same period of time to the stressor (fig. 2B; lanes 5 and 6). Importantly, this effect is reversed by exposure to rapamycin (a TORC1-specific inhibitor) just before the start of the stressing treatment (fig. 2B; lane 12), supporting that the observed effect of improved recovery from ER stress is TORC1 dependent. We conclude that insulin signaling via TORC1 attenuates IRE1 activity and promotes ER homeostasis. These observations are also consistent with the fact that InR deficient cells also exhibit increased IRE1-dependent accumulation of the XBP1*-*EGFP reporter (fig. 1C). We then performed further experiments to characterize the effect of downregulating these different signaling nodes on the dynamics of recovery of ER homeostasis. To this end, we transiently challenged with TM cells transfected with different RNAi, and then allowed them to recover in fresh medium over time. Importantly, downregulation of Raptor or SREBP (lanes 7, 8, 11 and 12), but not Rictor (see lanes 15 and 16), significantly delayed the recovery dynamics of cells from ER stress upon withdrawal of the ER stressor (fig. 2C), further supporting a significant role for TORC1 signaling for the maintenance of ER homeostasis and the regulation of UPR.

**Figure 3:**
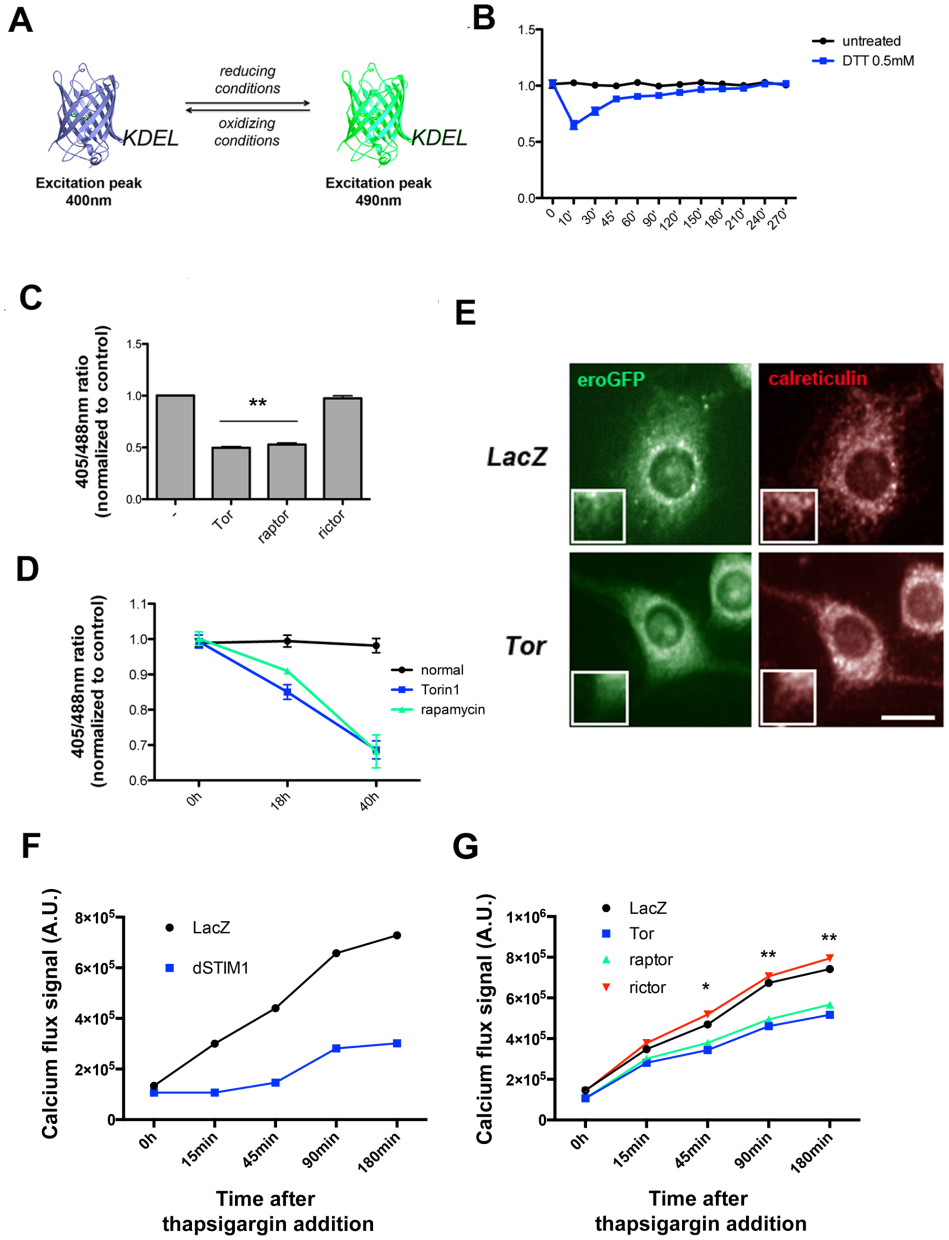
Sustained inhibition of TORC1 signaling leads to significant alterations in the ER luminal physicochemical properties in *Drosophila* cells. *(A)* S2R+/eroGFP is an inducible, stable cell line derived from *Drosophila* Schneider cells that conditionally expresses the ER-localized eroGFP reporter. The cartoon summarizes the spectral properties of the probe depending on the red/ox environment. *(B)* Exposure to the reducing reagent dithiothreitol (DTT; 0.5 mM) transiently decreases the ratio between oxidized (ex. 405 nM) and reduced (ex. 488 nM) forms of eroGFP [left panel] *(C and D)* S2R+/eroGFP cells were either transfected for 96 h with the indicated dsRNAs [C] or exposed to TOR inhibitors and measured at indicated times and concentrations [D], and 405/488 intensity ratios were computed. A minimum of ∼1200 cells were measured from four experimental replicates in three independent experiments. *(E)* S2R+/eroGFP cells transfected with either mock or TOR-targeted dsRNA were immunolabelled with an ER marker (anti-calreticulin antibody) and imaged using a spinning disk confocal microscope. Insets depict details from boxed zones. Scale bar: 20 µm. *(F and G)* S2R+ cells were transfected as indicated for 96 h and stained with Fluo-4 (MolecularProbes) for 30 min in fresh complete Schneider's medium. After obtaining a basal readout, cells were exposed to 1 µM thapsigargin, and fluorescence was monitored at the indicated times. Results plotted are the averages from 4 experimental replicates. Where indicated, statistical significance was assessed by applying t-Student's test. *: *p*<0.05; **: *p*<0.01; *n.s.*: non-significant

We reasoned that, if TORC1 has an important role in promoting ER homeostasis, inhibition of TORC1 signaling might affect key aspect of ER function, such as red/ox state or the maintenance of Ca^2+^ gradients. We thus monitored these luminal physicochemical properties in different backgrounds in S2R+ cells. We first estimated ER red/ox conditions in a *Drosophila* stable S2R+ cell line that conditionally express an ER-localized roGFP variant (eroGFP) (fig. 3A; [26]). The excitation peak of the reporter is dependent on the red/ox state of the ER lumen, and decreases in the ratio of signal obtained at 400 nm (oxidized species) as compared with that derived at 490 nm (reduced species) suggests a reducing environment exists at the ER lumen, which could hamper protein folding. While exposure to DTT only transiently decreaseed the 400∶490 (fig. 3B), depletion of either TOR or Raptor resulted in a ∼2-fold sustained decrease as compared with wild-type cells (fig. 3C). Long-term treatment of S2R+ cells with either rapamycin or Torin-1, inhibitors of TORC1 or both TORC1 and TORC2 respectively, also resulted in a significant progressive reduction of the eroGFP reporter (fig. 3D). These observations are consistent with our observation that TOR depletion leads to higher ratio of oxidized to reduced roGFP protein using non-reducing SDS-PAGE (fig. S2B). Although notable changes of roGFP red/ox ratios have been previously associated with ectopic distribution of the reporter under specific experimental conditions [27], we did not observe significant mislocalization of the reporter in TOR-deficient cells as analyzed by confocal microscopy (fig. 3E). Thus inhibition of TORC1 alters the red/ox conditions in the ER in a manner that presumably leads to lower rates of protein folding.

We also examined the ability of S2R+ cells to maintain Ca^2+^ homeostasis following depletion of TOR, Raptor, or Rictor. We used the fluorescent reporter probe Fluo-4 to assess the relative influx of calcium to the cell cytoplasm upon inhibition of the sarco/endoplasmic reticulum Ca^2+^-ATPase (SERCA2) calcium pump by thapsigargin. Upon inhibition of Ca^2+^ sequestration by addition of thapsigargin, an increase in the Fluo-4 signal can be readily observed in normal cells. However, this response is severely impaired in cells depleted of the fly homolog of Stromal Interaction Molecule 1 (STIM1), an ER-resident major regulator of calcium release-activated calcium modulator 1 (CRACM1/ORAI) channels (fig. 3F) [28]. Depletion of TOR and Raptor, but not Rictor, resulted in a moderate but significant relative decrease in Ca^2+^ flux upon disruption of the calcium gradient generated by SERCA2 (fig. 3G). We interpret that sustained suppression of TORC1 signaling induces significant alterations in physicochemical conditions in the ER lumen and/or membrane aberrancies, and these in turn disrupt ER-mediated Ca^2+^ homeostasis.

### TORC1 regulates ER morphogenesis

**Figure 4:**
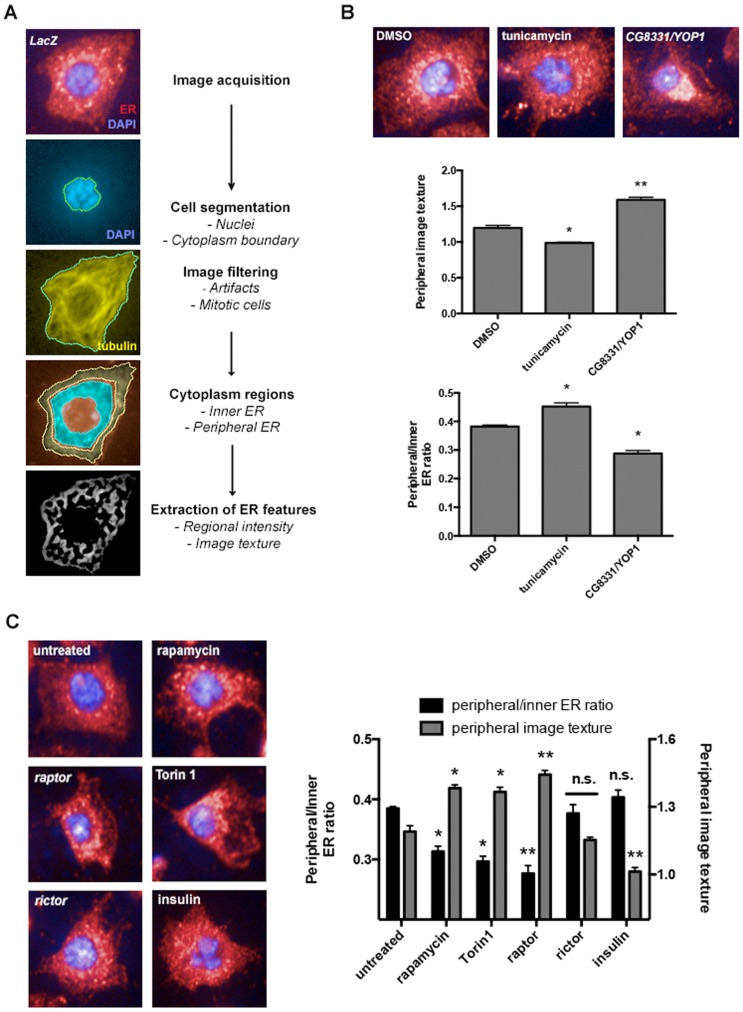
Inhibition of TOR-dependent signaling is associated with alterations in ER architecture and defective ER remodeling in *Drosophila*. *(A)* Flowchart summarizing the quantitative image analysis approach used. Images are representative examples of segmentation on the corresponding steps on the chart, applied on the same optical plane. *(B)* The image analysis procedure was tested on conditions that alter ER architecture, such as acute ER stress upon tunicamycin exposure (2.5 µg/ml, 8 h) or RNAi-mediated depletion of the structural protein CG8331/dYOP1. Data is sourced from three independent experiments with four experimental replicates including a minimum of ∼1200 cells. Panels show representative examples of cells from the indicated conditions and show the endoplasmic reticulum (red) with the overlayed image from DNA counterstaining (blue). Scale bar: 20 µm *(C)* ER structural integrity requires functional TORC1 signaling. Image presentation is represented as in [B]. Rapamycin (100 nM), Torin-1 (250 nM) and insulin (500 nM) treatments were applied for 24 h. Scale bar: 30 µm. Where indicated, statistical significance was assessed by applying t-Student's test. *: *p*<0.05; **: *p*<0.01; *n.s.*: non-significant.

One explanation for the observed alterations of ER homeostasis upon disruption of basal TOR signaling could be that TOR is essential for the structural integrity and size regulation of the ER. To test this hypothesis we first developed an image-based assay to estimate relative ER content and spatial distribution in S2R+ cells (fig. 4A). Following standard procedures, cells were seeded before fixation on concanavalin A-coated plates to promote cell spreading and facilitate imaging of the ER [29], [30]. To specifically label the ER, we used anti-calreticulin antibody, which yielded a closely matching pattern when compared with a chemical BODIPY-conjugated probe that specifically stains the early secretory apparatus (fig. S3A); or with the ER-localized reporter eroGFP (see fig. 3). Automated image analysis procedures were implemented to identify, from each cell, the cytoplasm boundary and to divide the cytoplasmic region into inner and peripheral sub-regions, which are normalized to the total cytoplasm area (fig. 4A). The extent of ER expansion is inferred by the ratio of ER intensity in the peripheral regions to the inner region. Induction of acute ER stress through exposure to TM (which leads to ER expansion), or depletion of an essential ER scaffold protein, such as CG8331/YOP1 (which led to ER collapse in S2R+ cells) resulted in significant increases or decreases respectively in the peripheral:inner ER ratio. (fig. 4B). Other features, such as those describing image texture, which captures the shape of the ER, were also measured (fig. 4A and B; see Methods; fig. S3C). Importantly, changes in ER distribution upon exposure to TM as determined using image-based methods correlated well with measurements of total cellular ER content as determined by flow cytometry (fig. 4B; fig. S3B). These data support that our quantitative image analysis procedures are capable of describing significant changes in ER morphology and distribution in single cells.

To determine the contribution of TOR signaling to ER architecture, size and expansion, we first analyzed cells undergoing prolonged exposure to rapamycin or Torin1. These treatments resulted in a “collapsed” ER and lower total ER content, as compared with untreated S2R+ cells (fig. 4C; fig. S3D and E), which also correlates with increased UPR signaling observed in in these cells (see fig. 2A). Importantly, depletion of the TORC1 complex constitutive component Raptor also led to collapse of the ER, while Rictor RNAi had no significant effect on ER content or structure (fig. 4C). Of note, insulin stimulation provoked opposite changes in the structure of the peripheral ER as estimated through image texture analysis (fig. 4C; fig. S3D and E). Taken together these data show that TORC1 activity is required to maintain ER distribution and shape in proliferating cells.

### SREBP is an essential regulator of ER homeostasis downstream of TORC1

**Figure 5:**
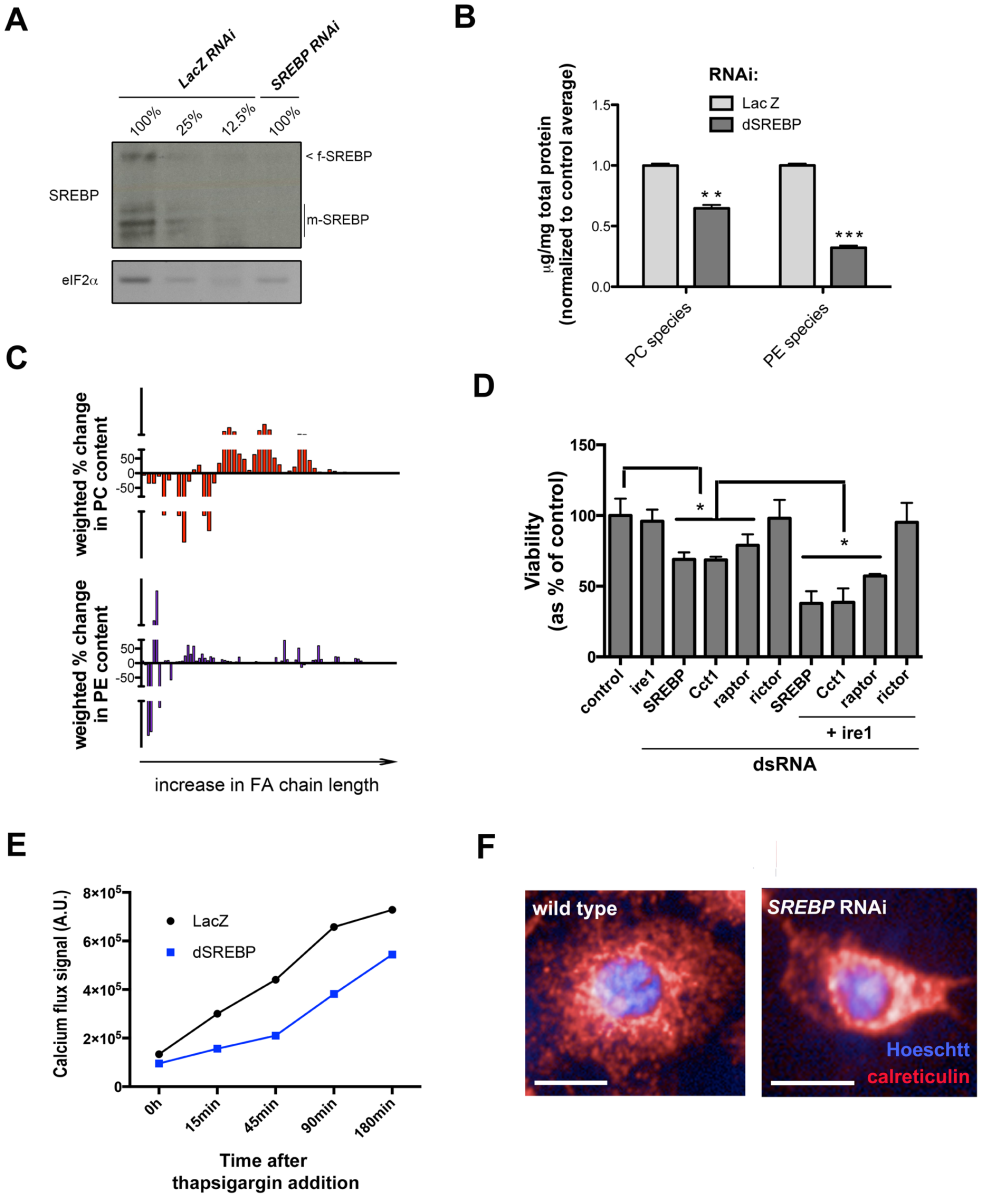
SREBP is an essential regulator of ER homeostasis in Drosophila and exerts its activity downstream of TORC1. *(A)* Western blot analysis for the efficiency of knockdown of SREBP protein in RNAi-transfected cells, using the 3B2 monoclonal antibody. f-SREBP, full-length SREBP protein (∼115 kD); m-SREBP, cleaved, mature N-terminal fragment of SREBP (∼78 kD) *(B and C)* Mass spectrometry analysis of PC and PE species from the lipid fraction of control and SREBP-depleted cells. Significance was calculated from three replicates, using t-Student test. **: *p<*0.005; ***: *p*<0.001. The right panel represents the fold-change in the relative content of specific species (*log()*) as compared between both conditions (table S5), with the *X* axis representing increasing chain length and unsaturation degree. *(D)* Cells transfected with the indicated single or double dsRNA treatments were assayed for viability under normal culture conditions. Viability measures are normalized to control (*LacZ* targeted RNAi) cells. Values are sourced and averaged from 4 biological replicates, and analyzed from two experimental replicates. *: *p*<0.05. *(E)* Calcium flux analysis of control and SREBP-depleted cells, as performed in fig. 3. *(F)* Representative confocal images of S2R+ cells transfected with the indicated dsRNAs and stained for chromatin (blue, DAPI) and ER compartment (red, α-calreticulin). Scale bar: 30 µm.

In addition to TOR, we identified the transcription factor SREBP, a known target of TOR signaling, as a potential regulator of ER homeostasis. We also isolated a number of other known regulators of phospholipid metabolism in our primary screen [24], [31]–[36]. Efficient RNAi-mediated depletion of SREBP (fig. 5A) caused significant increases in IRE1 activity and ER stress (fig. 1C, 2A). In accordance with previous studies, we observed that depletion of SREBP results in a marked decrease in the relative content of PC and PE (fig. 5B) [37], [38]. Detailed analysis further revealed aberrant fatty acid chain composition (fig. 5C). Because phospholipid metabolism is a direct driver of ER expansion in eukaryotes [10], [11], we reasoned that a primary effector of TORC1 signaling on ER homeostasis could be SREBP. In order to establish if SREBP is directly involved in ER homeostasis in *Drosophila* cells, we first determined if SREBP, and/or Cct1, an effector of SREBP, genetically interact with IRE1. Depletion of Raptor, SREBP, or Cct1 resulted in mild (ranging from 14 to 27%) decreases in cellular viability, and all three RNAi treatments were synthetically lethal with IRE1 (fig. 5D). Rictor did not exhibit synthetic lethality with IRE1 (fig. 5D). Of note, we did not observe a genetic interaction between SREBP and genes encoding members of the other two branches of the UPR (fig. S4E). Consistent with a role for SREBP in ER homeostasis, *Drosophila* SREBP protein is activated by endomembrane cleavage, and downstream transcriptional targets, such as Fasn or Sk1, are up-regulated in response to acute ER stress (fig. S4A–C).

We hypothesized that the TORC1-SREBP axis promotes ER homeostasis by regulating lipid biogenesis and/or mobilization. SREBP-deficient cells also phenocopy TORC1-deficient cells, as Ca^2+^ homeostasis (fig. 5E) and ER morphogenesis (fig. 5F) are disrupted in a similar fashion in these cells. Importantly, TORC1 signaling is required not only for the basal activity of SREBP, but also for its up-regulation during acute ER stress induction, as rapamycin significantly inhibited both intra-membrane cleavage of SREBP (fig. S4D) and its transcriptional output (fig. S4C) in both untreated cells, as well as in cells challenged with TM. We conclude that SREBP is a TORC1-dependent regulator of ER homeostasis in *Drosophila*.

**Figure 6:**
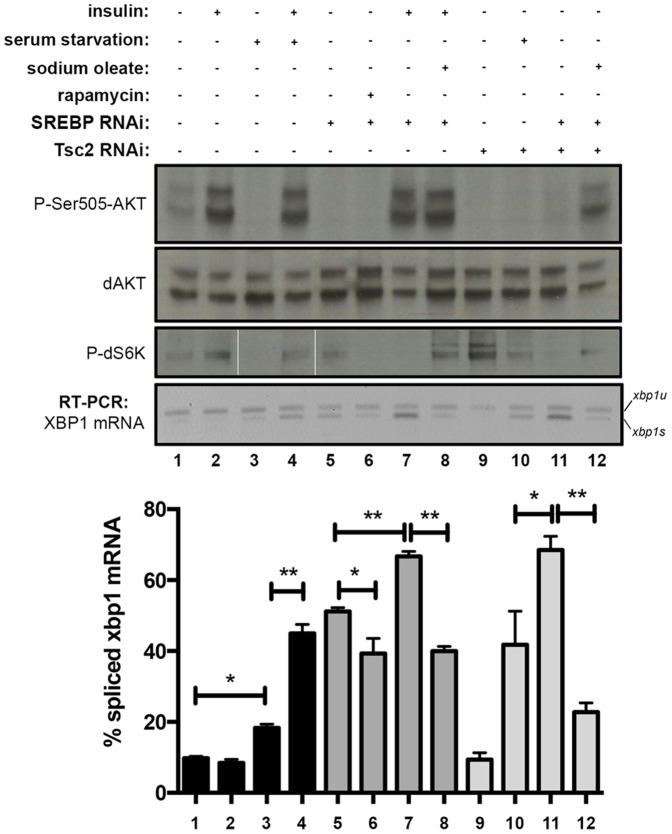
S2R+ cells were transfected for ∼96 h with the indicated dsRNA preparations, subjected to the indicated culturing conditions for ∼20 h, and harvested and RNA-extracted for RT-PCR analysis. Data was sourced from two independent experiments, each containing two technical replicates. Western blot analysis of analogous samples for P-AKT, total AKT and P-S6K relative levels is shown. Oleate rescue (0.5 mM, 6 h) and rapamycin rescue (100 nM, 1 h) treatments were applied as indicated. Statistical significance was assessed using t-Student's test. *: *p*<0.05; **: *p*<0.01; ***: *p*<0.005.

In addition to the novel role we have described for TORC1 as promoter of ER homeostasis, it is well established that TOR signaling promotes general anabolism, including protein synthesis, which could lead to ER stress [24]. For example, TOR overactivation, as occurs through suppression of the TORC1 regulator complex TSC1/2, has been previously suggested to be a source of ER stress, presumably because of the increased production of ER client proteins [39]. In order to determine if TORC1-mediated ER homeostasis is dominant over TORC1-mediated anabolism, we compared three genetic backgrounds using RNAi: control versus TSC2 deficiency or SREBP deficiency, and subjected these different backgrounds to a number of specific growth conditions, such as reduced availability of lipid precursors in the environment or insulin stimulation. We found that in our system, TSC2 deficiency (i.e. TORC1 sustained hyperactivity) only led to increased ER stress in conditions that imply limited availability of lipid precursors, such as serum starvation or concomitant SREBP depletion through RNAi (fig. 6; lanes 10 and 11). Conversely, SREBP-deficient cells exhibited a moderate decrease in ER stress when TORC1 signaling was suppressed (fig. 6; lane 6), and ER stress upon stimulation of TOR signaling (such as TSC suppression or insulin exposure; fig. 6, lanes 11 and 7, respectively). Importantly, exogenous supplementation of unsaturated fatty acids significantly attenuated the degree of ER stress in such conditions (fig. 6, lanes 8 and 12) further supporting an interpretation that lipid precursors are required to sustain ER homeostasis in proliferating cells (see also below). Taken together these results demonstrate that the role of TORC1 in promoting ER homeostasis predominates over TORC1-driven ER stress.

### Multiple Signals Converge on SREBP to Promote ER Homeostasis

**Figure 7:**
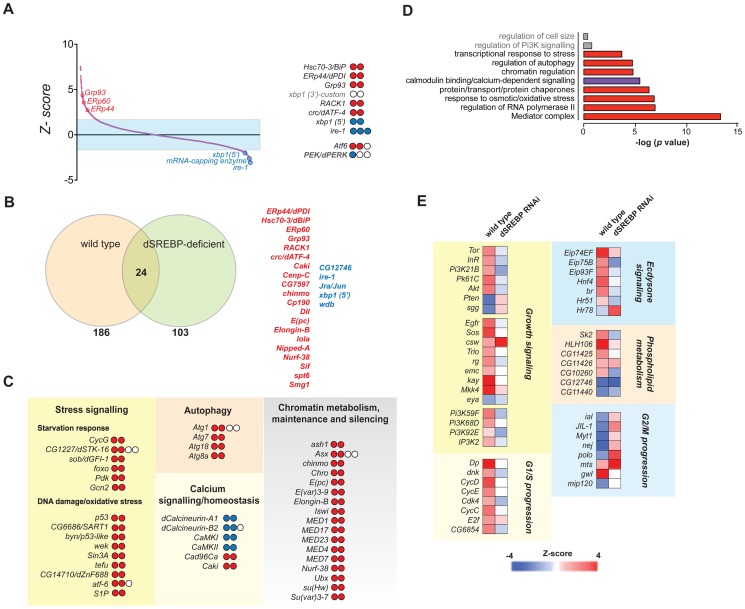
An *SREBP*-sensitized double RNAi screen for regulators of IRE1 activity highlights the functional interdependence between lipid metabolism and other pathways regulating ER homeostasis. *(A)* Distribution of ranked Z-scores for all interrogated genes. Core ER-chaperones and ER regulators map beyond the fixed threshold, demonstrating that in this background the system is sensitive for major regulators of ER homeostasis. The right panel depicts positive (red) and negative (blue) hit amplicons for the listed genes. *(B)* Diagram representing the relatively poor overlap between both screens, mainly consisting on core ER function and UPR signaling regulators as listed in the left panel. *(C)* Major functional categories and inclusive hits found in the SREBP-sensitized screen. Positive and negative-scoring amplicons are represented in red and blue respectively. *(D)* GO enrichment analysis for the hit lists obtained. Red denotes categories with predominantly positive hits, and blue denotes categories with predominantly negative hits. Two major GO categories detected for the wild-type background screen, relatively underrepresented upon SREBP downregulation, are depicted in grey. *(E)* Comparison of scores between both backgrounds for the main functional categories identified as ER homeostasis regulators in the wild-type background screen.

We next sought to determine if the other genes and functional modules identified in our original screen contribute to ER homeostasis and lipid mobilization in a manner dependent or independent of SREBP. Thus we performed a sensitized RNAi screen using the same dsRNA sub-libraries used for XBP1-EGFP splicing, in cells simultaneously RNAi-depleted of SREBP (fig. 7A). Depletion of ER homeostasis regulators that function independently of SREBP signaling should further increase or decrease XBP1-EGFP splicing. Conversely, genes functioning as part of an SREBP-dependent pathway are unlikely to further exacerbate the phenotype of SREBP deficiency. For example, depletion of the chaperones Hsc70-3/BiP and Grp93, or the protein disulfide isomerases ERp60 and ERp44, which mediate protein folding and not lipid biogenesis directly, further increased ER stress in SREBP-deficient cells, while depletion of TOR did not (fig. 7A and B). In fact, the two screens shared only 24 hits, mostly comprised by such core ER function regulators, while approximately ∼89% primary hits from the wild-type background screen were not isolated in the SREBP-sensitized screen (fig. 7C). Importantly, GO terms such as “AKT signaling” or “phospholipid biosynthesis”, which are enriched in the list of hits following a screen of unsensitized cells, are not similarly enriched in a hits lists following the same screen of SREBP*-*deficient cells (fig. 7D). Accordingly, RNAi targeting genes encoding proteins that we predict to functionally interact with SREBP based on their orthologs, such as the transcription factors Hnf4, Hr48, Hr51, Eip74EF and Eip75B [40], [41], were associated with significant IRE1 activation in wild type cells (fig. 1), but do not lead to further increases in XBP1-EGFP splicing in SREBP-deficient cells (fig. 7E). Similarly, depletion of *bona fide* SREBP-target genes such as Sk1/2, CG11425 and CG11426 does not further exacerbate the phenotype of *SREBP*-deficient cells (fig. 7E). Thus, we conclude that a major proportion of genes contributing to ER homeostasis as determined by our primary screen (fig. 1), including positive growth regulators (TOR, Egfr, Sos, AKT, Pk61C) and G1/S progression regulators (CycD, Dp, CDK4) are epistatic to SREBP.

We identified five major classes of genes whose depletion significantly up-regulates ER stress specifically in SREBP deficient cells: i) genes involved in the starvation response, such as *foxo*; ii) genes involved in general stress responses and survival; iii) genes regulating autophagy; iv) genes involved in calcium homeostasis; and v) genes involved in chromatin remodeling (fig. 7C). Thus, stress response pathways, including the IRE1-XBP1 branch of the UPR, are activated in SREBP-depleted cells to help buffer the levels of ER and cellular stress resulting from lipid and glycerophospholipid imbalance, rather than directly aiming to restore phospholipid levels [14].

### TORC1 and SREBP-mediated ER homeostasis is dependent on Lipid/Phospholipid Metabolism

**Figure 8:**
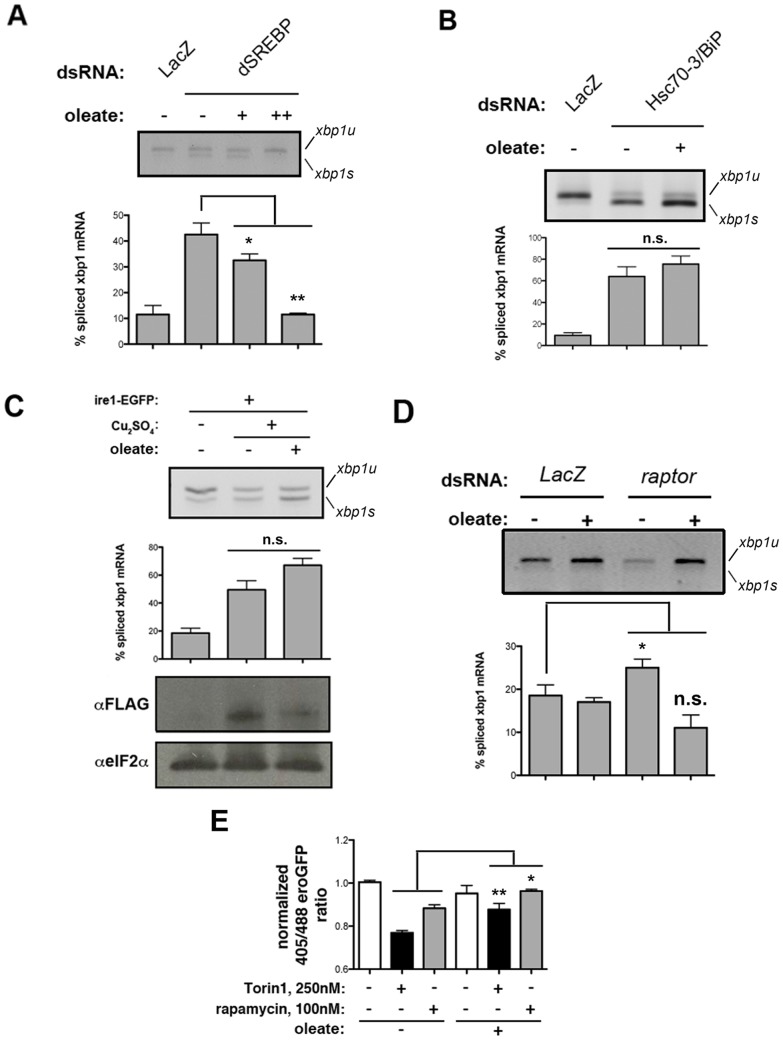
Unsaturated free fatty acids specifically rescue ER stress and deficient UPR attenuation associated with alterations in TOR signaling and SREBP activity. *(A)* S2R+ cells were transfected with SREBP-targeting dsRNA, and cultured in normal conditions or further supplemented with 0.1 mM (+) or 0.5 mM sodium oleate (++) for 6 h. Total RNA was extracted and splicing of endogenous XBP1 mRNA was analyzed by RT-PCR. Three independent experiments, each containing two technical replicates, were analyzed. *(B)* S2R+ cells were transfected with Hsc70-3/BiP-targeting dsRNA and cultured in normal conditions (-) or further supplemented with 0.5 mM sodium oleate (+). Splicing of endogenous *XBP1* mRNA was assessed by RT-PCR. Three independent experiments, each containing two technical replicates, were analyzed. *(C)* Stable S2R+ cells conditionally expressing an IRE1-EGFP construct from a metalothionein promoter were cultured in normal conditions or supplemented with 500 mM Cu_2_SO_4_ for 24 h to achieve robust expression of the heterologous construct. Induced cells where further supplemented with 0.5 mM sodium oleate (+) for 6 h or not supplemented. Splicing of endogenous *XBP1* mRNA was assessed by RT-PCR. Three independent experiments, each containing two technical replicates, were analyzed. *(D)* S2R+ depleted of Raptor by RNAi were supplemented for 6 h with 0.5 mM sodium oleate as indicated. Splicing of endogenous *XBP1* mRNA was assessed by RT-PCR. Significance beacons denote comparison with control, untreated cells. Three independent experiments, each containing two technical replicates, were analyzed. *(E)* S2R+/eroGFP cells were exposed to the indicated inhibitors of TOR signaling for 24 h, supplemented for 6 h with 0.25 mM sodium oleate as indicated, and analyzed by quantitative imaging to estimate their ER luminal red/ox coefficient. A minimum of ∼1200 cells were analyzed per condition in three independent experiments. Where indicated, statistical significance was calculated applying t-Student's test *: *p*<0.05; **: *p*<0.01; *n.s.*: non-significant.

We reasoned that if the TORC1-SREBP signaling axis promotes ER homeostasis by positively regulating lipid metabolism, especially fatty acid mobilization and phospholipid synthesis, exogenous supplementation of lipids should suppress the activation of IRE1 that is due to disruption of lipid biogenesis. To perform rescue experiments, we used Na-C18∶1 (sodium oleate), because this unsaturated fatty acid efficiently restores viability in flies with genetic deficiency in SREBP activity [38]. We observe that exogenous oleate rescues the effects of SREBP depletion on IRE1 activity in S2R+ cells (fig. 8A). Interestingly, oleate does not suppress IRE1 RNAse activity associated with depletion of Hsc70-3/BiP (fig. 8B), nor overexpression-associated constitutive activation of IRE1 (fig. 8C), suggesting that the relieving effect of oleate on UPR signaling is likely specific to defects in lipid metabolism, and not due to a direct effect on the ability of IRE1 to sense unfolded peptides and/or lipid composition of the ER membrane [42]. Importantly, alterations in ER luminal red/ox conditions and UPR activity caused by sustained TOR inhibition are also reversed by exogenous supplementation of oleate (fig. 8D and E). These experiments demonstrate that TOR-SREBP activity promotes ER homeostasis through the positive regulation of lipid biogenesis and/or mobilization.

### Defects in intracellular lipid mobilization correlate with altered ER homeostasis

**Figure 9:**
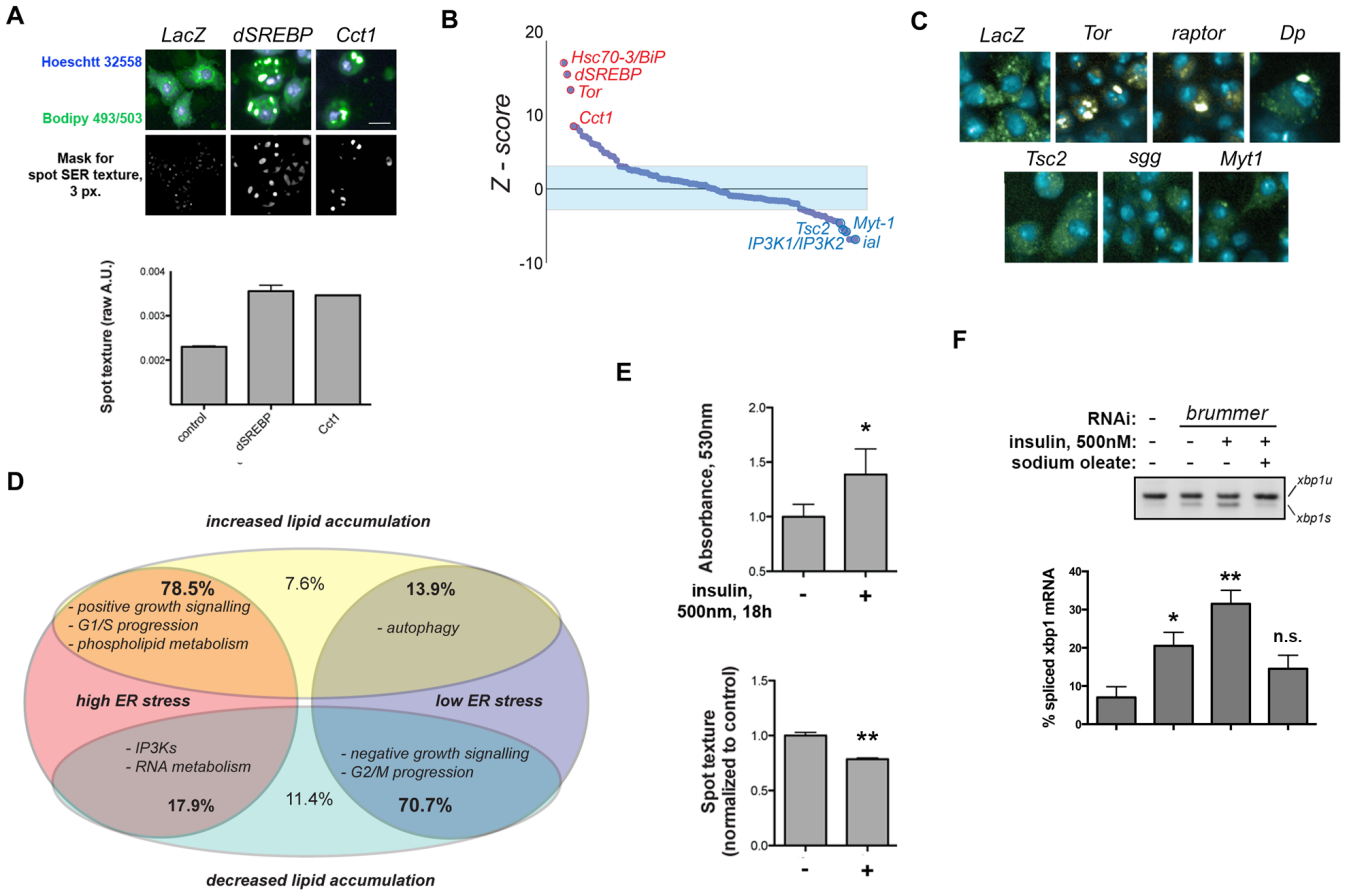
Impact on ER homeostasis from the hits identified in the XBP1-EGFP screen correlates with altered lipid distribution and mobilization in the cell. *(A)* Demonstration of the image-based assay used to monitor lipid distribution in S2R+ cells. S2R+ cells were transfected for ∼96 h with the indicated dsRNAs, stained for neutral lipids and DNA, fixed and imaged (upper row). A corresponding image of the texture mask used to estimate the relative distribution of lipids is also shown (lower row). *(B)* Distribution of ranked Z-scores for interrogated genes (XH set). *(C)* Exemplary images for indicated RNAi treatments. *(D)* Diagram representing the distribution of interrogated genes through 4 different major classes, according to their impact on XBP1-EGFP splicing and differential neutral lipid distribution. Prominent functional classes for each group are denoted. *(E)* The image-based readout [bottom panel] for lipid distribution in the cell recapitulates mobilization observed upon stimulation with insulin (1 µM, 18 h) as estimated by free glycerol release [top panel] from S2R+ cells. *(F)* RNAi-mediated depletion of the triacylglyceride lipase Brummer in S2R+ cells results in ER stress, which is increased upon stimulation with insulin. Total RNA was prepared and XBP1 mRNA processing was assessed through RT-PCR. Two independent experiments, each including two replicates, where analyzed. Where indicated, statistical significance was calculated applying t-Student's test *: *p*<0.05; **: *p*<0.01; *n.s.*: non-significant.

To confirm the hypothesis that TORC1-SREBP integrates diverse signals to regulate lipid metabolism or mobilization, we tested whether depletion of hits identified in our primary IRE1 screen affects lipid biogenesis and/or mobilization. We thus screened a curated sub-library of hits and related genes (the “XH set”; see Table S3) to assess the impact of depleting our identified ER regulators on the distribution of storage lipids in the cell, using quantitative image analysis of non-polar lipid staining [43]. Wild-type S2R+ cells growing in normal medium exhibited a mostly diffuse cytoplasmic distribution of lipid staining, with small punctate structures (fig. 9A, left upper panel). Depletion of SREBP or Cct1 provoked a marked accumulation of lipids in large, bright structures (fig. 9A, middle and right upper panels), as has been previously shown as a consequence of imbalances in PC and PE levels [43], [44]. To quantify lipid distribution, we developed image analysis approaches, which largely capture these features, such as speckle-like image texture (fig. 9A, lower panels; Methods, and fig. S5A). Importantly, depletion of genes that increase XBP1-EGFP splicing also very often increased lipid droplet accumulation (fig. 9B and C; see also Table S2). For example, depletion of TOR, Raptor, and Dp leads to the generation of enlarged, intensely stained cytoplasmic inclusions (fig. 9C). Conversely, inhibition of TSC2, GSK3B, and Myt1, which yielded significantly lower activities for the XBP1-EGFP reporter, decrease the size of cytoplasmic lipid deposits (fig. 9C). In fact, for ∼70% of the XH set, increases or decreases in XBP1-EGFP splicing directly correlate with increases or decreases in lipid droplet size (fig. 9D).

Our data suggest that insulin upregulates SREBP signaling through TORC1 to ensure phospholipid supply and ER homeostasis, at least partly through the mobilization of intracellular stores. Indeed, upon prolonged stimulation with insulin, wild-type *Drosophila* S2R+ cells liberate free fatty acids from intracellular stores as monitored both through our image-based assay and classical biochemistry (fig. 9E). Our data clearly shows that TORC1-dependent growth signaling is coupled to the positive control of lipid mobilization, in agreement with numerous previous reports of phenotypes associated with genetic ablation of key regulators of insulin signaling in a conserved fashion [45], [46]. A requirement for lipid mobilization to sustain cell growth upon insulin stimulation is further supported by the fact that RNAi-mediated depletion of the ATGL1 homolog Brummer stimulated IRE1 activity, which was further exacerbated upon insulin stimulation (fig. 9F). Taken together, these observations support a model whereby cell growth and proliferation require the mobilization of lipid stores for the synthesis *de novo* of endomembranes through the coordination of growth signaling and ER homeostasis surveillance pathways.

### The ER homeostasis network

**Figure 10:**
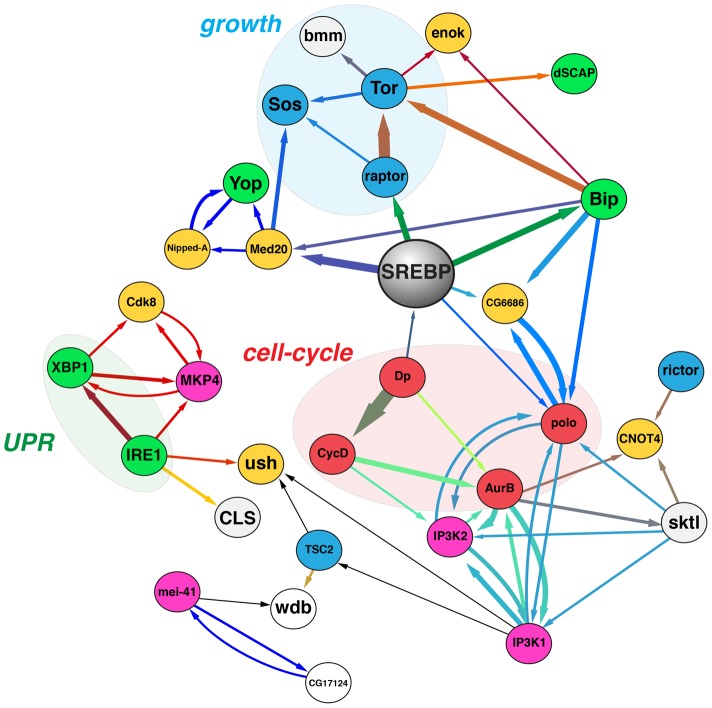
A number of major signaling pathways, including a core module regulating G1/S transition in proliferating cells, converges on TORC1/SREBP signaling to regulate ER homeostasis. The figure shows a graph depicted after Hierarchical Interaction Score analysis of the phenotypes displayed by ER homeostasis regulators across functional assays. The direction of each edge represents functional dependencies, and the thickness of each line defines the strength of each dependency. Nodes are coloured based on the proteins known function or physiological role as follows: *blue* = regulators of growth; *red* = regulators of cell-cycle progression; *green* = ER proteins; *yellow* = regulators of transcription/translation; *grey* = regulators of lipid metabolism; *pink* = stress response proteins; *white* = general signaling proteins.

To obtain a systems-level view of the networks regulating ER homeostasis, we compiled scores for each gene in the XH set across different assays (XBP1-EGFP reporter, eroGFP reporter, lipid distribution and cell size) and in different conditions (table S4) to generate a multidimensional phenotypic signature for each gene. We then generated a graph of functional dependencies amongst these genes using the Hierarchical Interaction Score (HIS) analysis [47]. Notably, the directional edges generated by HIS do not necessarily represent classical enzyme-substrate relationships, but rather are representative of functional dependencies between genes. For example an edge with a direction from A to B, means that the phenotypes following depletion of B are a subset of the phenotypes that occur following depletion of A [47]. Only edges above a certain threshold are included (see Methods). Many proteins are part of highly interconnected modules, or sub-networks; that is, they exhibit more interactions with other proteins in the module than with proteins in other modules (fig. 10). Two prominent sub-networks can be distinguished in this graph: one that regulates cell growth and includes Raptor, TOR, and Sos; and another that regulates cell-cycle progression and includes polo, AurB, Dp, and CycD. Critically, SREBP links these two sub-networks, and thus we propose that SREBP plays a key role in ensuring ER homeostasis in the context of coordinated cell growth and cell cycle progression.

**Figure 11:**
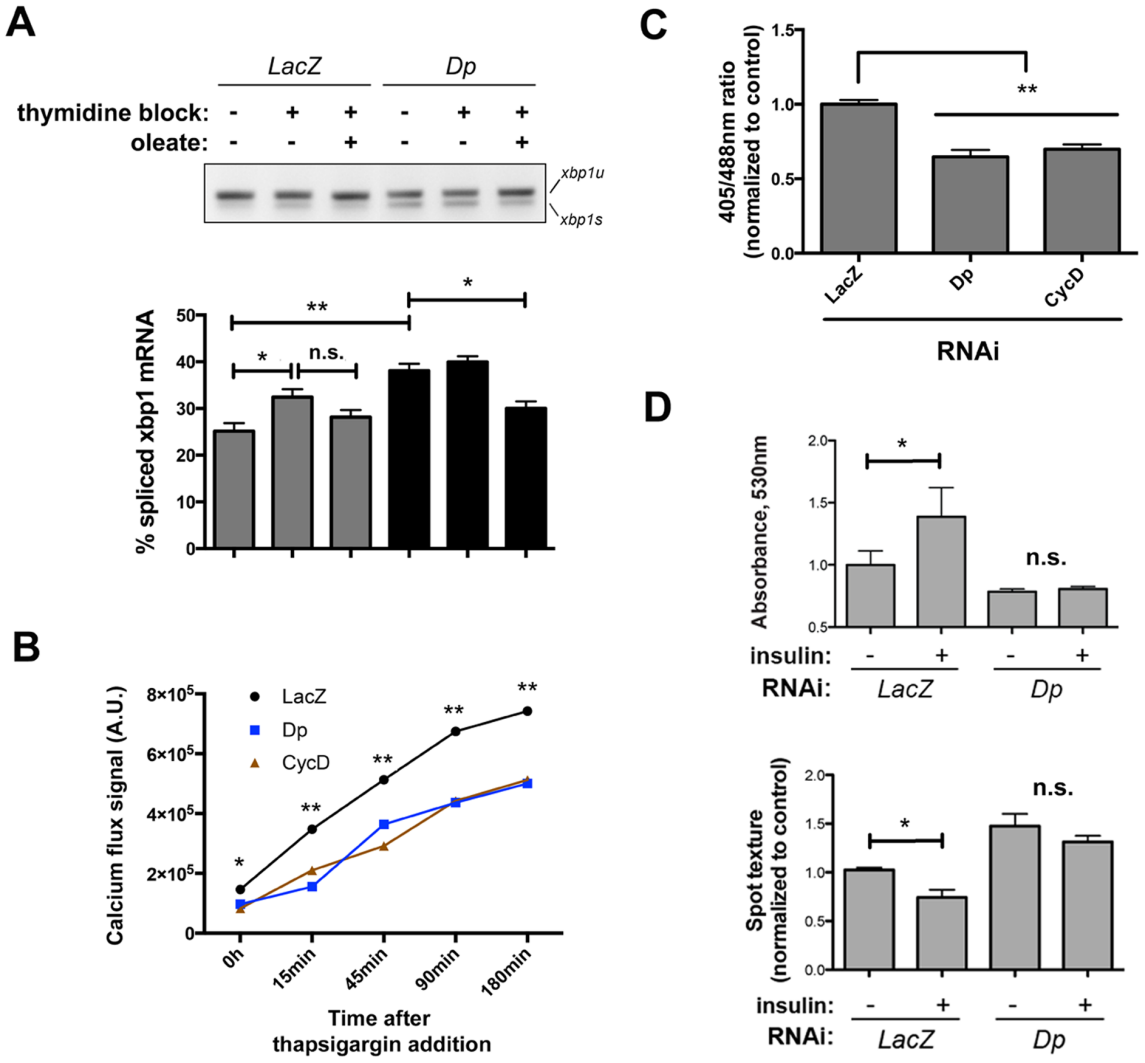
Validation of regulators of cell cycle G1/S progression as regulators of ER homeostasis through lipid metabolism. *(A)* Validation of the essential regulator of G1/S transition Dp as a modulator of ER homeostasis through lipid metabolism. The essential regulator of G1/S transition *Dp* was depleted from S2R+ cells by RNAi, exposed to the indicated conditions (oleate supplementation: 0.5 mM, 6 h), and RNA was extracted for the semiquantitative analysis of XBP1 mRNA splicing levels, as compared with non-specific RNAi treatment (LacZ). Three independent experiments, each containing two technical replicates, were analyzed. *(B and C)* Luminal physicochemical conditions are altered upon RNAi-mediated depletion of G1/S transition regulators. ER red/ox [C] and calcium mobilization [B] were monitored as described previously in cells transfected with the indicated dsRNAs. *(D) Dp* RNAi leads to defects in growth signaling-mediated lipid mobilization, as assessed by conventional biochemistry [upper panel] and quantitative image analysis [lower panel] as described in figure 7. Where indicated, statistical significance was calculated applying t-Student's test. *: *p*<0.05; **: *p*<0.01; *n.s.*: non-significant.

In the network model, SREBP is linked to Dp, which is turn is linked to CycD. CycD is a conserved promoter of Dp activity and G1/S entry [48], [49]; fig. S5A), and our data shows that depletion of these genes engages the UPR (fig. 1) – presumably as they are arrested at the G1/S transition. Notably, we have found that both Dp and CycD (as well as other G1/S progression regulators) are epistatic to SREBP as determined by double RNAi screening (fig. 7). Our network model further suggests that the engagement of the UPR following Dp or CycD depletion correlates with diminished SREBP activity (fig.10), and we sought to validate this prediction. While arresting cells at the G1/S boundary through thymidine treatment or RNAi-mediated depletion of Dp increases IRE1 activity, this effect could be rescued by exogenous oleate (fig. 11A). Moreover, depletion of Dp and CycD also significantly impacted ER luminal red/ox conditions and Ca^2+^ balance (fig. 11B and C) in a similar scale and direction as depletion of SREBP itself, or genes that promote growth such as TOR and Raptor (fig. 3 and 5). Finally, Dp RNAi depletion (fig 11D) or thymidine blockade (fig. S5B) significantly hampered intracellular lipid mobilization upon insulin stimulation. While the mechanisms by which G1 arrest leads to impaired lipid mobilization and loss of ER homeostasis in S2R+ cells remain to be characterized, these data support the model that SREBP-mediated lipid metabolism integrates signals emanating from the cell cycle control network in order to ensure ER homeostasis through the G1/S transition in a coordinated manner with cell growth.

## Discussion

We have conducted a series of unbiased, genome-scale RNAi screens to describe, for the first time, a signaling network that promotes ER homeostasis in proliferating cells. Based on the generation of a graph representing hierarchical functional interactions, it is clear that the signaling network that regulates ER homeostasis has a largely uneven topology (fig. 10). However, the architecture of this network suggests that a major proportion of the information flow from the modules that regulate ER homeostasis goes through the TORC1-SREBP axis. Thus functionally, TORC1 and SREBP represent a “central manager” in the ER homeostasis network. We propose that cells may have evolved such a network structure to ensure that ER function is regulated in a coordinated fashion with different cellular functions such as growth and proliferation, and that ER homeostasis is robust to signal fluctuations. TORC1 and SREBP might serve as a gatekeeping mechanism to promote lipid mobilization and changes in ER shape by integrating numerous upstream signals and being activated only once a certain threshold has been satisfied. This architecture thus wrests the control of lipid mobilization and biogenesis from independent modules, whose actions could potentially conflict, especially if they were spuriously or transiently activated. This model of the architecture of the ER homeostasis network is a systems-level finding that could not have been identified though conventional bottom-up approaches.

We propose that in normal conditions the branch downstream of TOR regulation controlling lipid anabolism prevents ER stress that could potentially occur due to increases in TOR-driven protein synthesis (fig. 6), and that constitutive activation of TOR signaling leads to ER stress only when phospholipid availability (or that of required precursors) is limiting. Indeed, markers of sustained ER stress are elevated in inner regions of tumoral lesions of *TSC2*-knockout mice [39] (which, as solid tumors, often display reduced availability of nutrients and oxygen [50]), and prolonged, pharmacologically induced ER stress tends to suppress AKT/TOR signaling [8], [51]. However we found across our functional assays that TSC2-deficient cells display not only *lower* levels of XBP1-EGFP reporter activity or classical markers of ER stress, but also a higher degree of mobilization of intracellular lipid depots (figs. 6 and 9, and see below). Thus, while insulin/TOR-driven translation significantly increases the concentration of ER client proteins, ER homeostasis can be maintained as long as TOR also acts to ensure phospholipid availability through the up-regulation of SREBP. As a related example, a recent study on T-cell activation and proliferation found a clear requirement of Pi3K/TOR-dependent activation of SREBP for survival and metabolic homeostasis [52]. Thus, conditions where TOR-mediated translation overwhelms TOR-mediated ER homeostasis lead to robust IRE1 activation. The impact of sustained TORC1 inactivation on ER homeostasis should also be taken on account when interpreting the phenotype of TORC1 downregulation in other functional screens, especially those studying aspects of cell physiology closely related to ER function.

Whereas inhibition of TOR has been thought of as a therapeutic target in metabolic diseases that course with ER stress by decreasing ER client load and increasing insulin sensitivity (by for example, blocking TOR-mediated phosphorylation of IRS1) [51], our results demonstrate a requirement for TOR signaling in promoting ER expansion and homeostasis in eukaryotes. Thus targeting TOR signaling as a therapy for the treatment of metabolic disease could potentially favor, and not limit, ER stress, potentially leading to undesired effects. Finally, an interesting open question is to what extent altered UPR signaling regulation contributes to the effects of sustained TOR inhibition on aging, as recent reports have suggested links between up-regulation of proteostasis maintenance mechanisms and extended lifespan [53].

## Materials and Methods

### Cell culture and reagents

S2R+ cells were grown in Schneider's medium (Sigma) supplemented with 10% fetal bovine serum (FBS; Gibco) and 1x Penicillin/Streptomycin (Gibco) unless stated otherwise, at 25°C. Copper sulphate, Hoeschtt 33258, tunicamycin (Tm), dithiothreithol (DTT), rapamycin and sodium oleate (Na-C18∶1) were purchased from Sigma. Torin-1 TOR kinase inhibitor was purchased from Tocris. Bodipy 493/530, antiGFP rabbit polyclonal antibody and Alexa-488 and Alexa-647 immunoconjugates were purchased from Molecular Probes (Invitrogen). Tables containing use, origin and characteristics for primary antibodies and oligonucleotides used in this study are provided below (Tables 1 and 2). Stable S2R+ cells conditionally expressing the eroGFP reporter or the IRE1-GFP construct were obtained by cotransfection with the pCoPuro plasmid in a 20∶1 ratio and passaging for >15 days in medium containing 5 µg/ml puromycin (Sigma) [54]. Subclones were assessed for expression, localization and integrity of the reporter. Anti dP-S6K and anti-dAKT were kindly provided by Sally J. Leevers (CRUK-LRI, London). Anti-dAKT serum was further purified following standard protocols; briefly, 0.5 ml affinity columns were prepared coupling SulfoLink activated resin (Thermo Scientific) to the synthetic peptide sequences STSTSLASMQ (total Akt) (GeneScript, USA). Serum was clarified by ultracentrifugation and passed manually three times at 50 µl/sec in the presence of 0.1% Tween20. After extensive washes in TBS-0.1% Tween20, specific antibodies were eluted in 0.3 M glycine pH 2.8, neutralized and dialyzed against TBS-10% glycerol for storage.

**Table 1:** Antibodies used in the study.

Antigen	Source	Use	Dilution
GFP	Invitrogen (Rb)	IF/WB	1∶10000/1∶4000
Calreticulin	Abcam (Rb)	IF	1∶2000
P-eIF2α (S51)	Enzo Biosc. (Rb)	IF/WB	1∶400/1∶2000
eIF2α	Abgent (Ms)	IF/WB	1∶100/1∶1000
α-tubulin	Abgent (rat)	IF	1∶1000
dSREBP (3B2)	Rawson lab (Ms)	WB	1∶1000
P-Ser505-AKT	Cell Signaling	WB	1∶2000
AKT	Leevers lab (Rb)	WB	1∶1000
P-S6	Leevers lab (Rb)	WB	1∶1000

**Table 2:** Oligonucleotides used in the study.

ID	Sequence (FWD)	Sequence (REV)
dXBP1 (spl. assay)	CAGATGCATCAGCCAATCCA	CACAACTTTCCAGAGTGAG
dFASN (qRT)	CAACCGCATCTCCTTCAC	CAGCACCACACATCCATC
dSK1 (qRT)	ATAGAGAGCGAGCGATTGAG	CCTTGCCCAGTAGATAGGAC
dG3PD (qRT)	CGGACCTGAAGAAGATGGTG	AGCACACGACGCATAATCTT
dActin (qRT)	CAGCACTTCGCATCAAGGCCCAAG	CACACACCAAGGCAGTGCTGTCC
*raptor* (qRT)	CGGAGATGGAGAGTGGAAAG	TTGGCGTTGGGATAAGGA
*rictor* (qRT)	CGGAGAGTTCGTTGTCCAGT	ACCAGCGGAGTTTGAAGTTG
dSREBP (qRT)	GCAGTTCCTTCGTTTTCTTTTC	GGCTTCCATTTCCAGTCAGTT
TOR (qRT)	GACGAGACGAACAGCAAAG	ACCTACCAAAACGGACACCA

### Reporter vectors

The parental vector pUASt-XBP1-EGFP was a kind gift from H Steller and HD Ryoo (NYU School of Medicine, USA) [55]. pMT-V5-eroGFP was obtained by cloning a *EcoRI/XhoI*-digested fragment encompassing the coding fragment of roGFP-HDEL from the p28M kar2ss-roGFP2-HDEL vector [26] into the pMT-His-V5 expression vector (Invitrogen). The inducible IRE1-EGFP construct pMT-IRE1-Flag-EGFP (fig. 6C) was obtained by cloning a PCR product from pcDNA5.1/3xFLAGhIRE1-EGFP [56] with flanking *KpnI* and *NotI* sites in the pMT-His-V5 vector.

### dsRNA synthesis, RNAi treatment and siRNA transfection

dsRNA synthesis for batch and RNAi screening library use was carried out using the MEGAScript T7 IVT kit (Ambion-Invitrogen) from T7 promoter-tailed PCR products, and purified on vacuum-driven 96-well filter plates (ThermoScientific). RNAi treatment through “bathing” for RNAi screening was performed as described previously [57] Batch transfection for dsRNA and DNA was performed using Effectene Reagent (Qiagen) following manufacturer's protocols. The following amplicons (www.flyrnai.org) were routinely used for validation and biochemical experiments: DRSC04743 (ATF6), DRSC36702 (PEK/dPERK), DRSC15606 (IRE1), DRSC36734 (TOR), DRSC18359 (Raptor), DRSC20071 (Rictor), DRSC37563 (SREBP), DRSC33319 (Hsc70-3/BiP), DRSC10502 (Brummer), DRSC08157 (Cct1), DRSC27133 (CG8331/YOP1), DRSC07402 (Dp), DRSC25031 (CycD), DRSC20158 (STIM1).

### RNA extraction, RT-PCR and qRT-PCR procedures

Total RNA was extracted using Trizol-chloroform (Invitrogen). Purified nucleic acids were digested at 37°C for 30 min with 10 U of recombinant DNAse and extracted again with standard phenol:chloroform procedures. ∼1 µg of purified RNA was converted to cDNA in a reaction containing 1 mM dNTPs, 0.02 mM DTT, 1 U MMLV, 2 U RNAse OUT (Invitrogen), and 0.3 mM random hexamers (Promega), 90 min at 37°C. A 20-fold dilution of the reaction product was normally used for conventional PCR and qRT-PCR analysis. Conventional RT-PCR analysis was resolved in TBE 0.5X/2% agarose gels, and quantified by densitometry. qRT-PCR analysis was carried out using the Precision MasterMix reagent (PrimerDesign, UK) following supplier's recommendations, on a 7300 AB system (Applied Biosystems). ΔΔCt quantitation procedure was used from absolute measurements from three technical replicates per sample.

### High-throughput sample processing, automated image acquisition and analysis for functional genetic screenings

All automated sample processing and liquid handling was performed on a Cell::Explorer station (PerkinElmer and ThermoScientific). After appropriate plating and incubation for ∼115 h of transfected cells in the presence of dsRNA, cells were fixed and permeabilized in PBSF (PBS1x, 4% paraformaldehyde, 0.1% Triton-X100) for 20 min at room temperature. After three washes in PBS, cells were blocked for 1 h in 2% BSA-PBS. Anti-GFP was then incubated (1∶10000) in a PBS-0.5%BSA-0.01%Triton-X100 solution overnight. After four washes, secondary antibodies were added and incubated for 90 min. Samples were washed twice in fresh PBS, and a 1∶5000 dilution of Hoescht 33258 was added for 30 min. Samples were finally rinsed in fresh PBS and sealed for acquisition.

Automated image acquisition and analysis for the *XBP1*-EGFP reporter-based screen was performed using an Opera HTS microscope, using a 20x objective and sequential excitation with 405 and 488 nm laser lines, and 450/540 CCD camera dicroic filters. 25 independent fields were imaged per well. Nuclear segmentation, intensity measurements, and image quality filtering and calculations were performed simultaneously to acquisition using a custom Acapella 2.5 image analysis script (PerkinElmer). The relative number of EGFP-positive cells was normalized to the control wells on the plate, and resulting values were averaged between the two replicates for each plate set. After ranking all datasets and calculating an average normalized value |x| and corresponding standard deviation using all control wells, a *Z*-score was obtained for each amplicon as Z_n_ = (|x|-a_n_)/σ. Based on the redundancy of the K/P set from our screening libraries, we considered a kinase or a phosphatase gene as a hit in our screen only when two or more amplicons surpassed a threshold of *Z*≥1.5 or *Z*≤-1.5, as previously described in analogous screening approaches.

For lipid staining, fly cells were fixed in 4%PFA-PBS and stained for 1 h with a PBS solution containing 1∶1000 Bodipy 493/530 and 1∶5000 Hoescht 33258 dilutions. Cells were rinsed once in fresh PBS and sealed. Imaging was performed using similar settings, using either a 20x/0.6A or a 40x/0.9A objective. Image collections were then uploaded to a remote server running Columbus 2.3 (Perkin Elmer). Image analysis consisted of (1) nuclear segmentation on the DNA stain channel, (2) cell segmentation on the lipid stain channel, (3) image quality filtering, and (4) estimates of lipid signal distribution, such as analysis of texture parameters at different pixel sizes on the lipid stain channel, or segmentation based on intensity mask an calculation of the relative signal content.

Live cells expressing the eroGFP reporter were imaged for emission at 510/540 nm upon excitation with 405 and 488 nm sequentially. Typically 10 fields (approx. ∼1000 cells) were acquired using a 20X/0.6A objective, and cell segmentation, image quality filtering, signal quantitation, range estimation for each channel and calculations were performed simultaneously using a tailored Acapella 2.5 script.

For ER distribution assessment, cells were batch-transfected in 24-well plates with the indicated dsRNAs for 96 h, or subjected with the indicated treatments. Subsequently, cells were harvested and transferred for 2 h to concanavalin A (Sigma)-coated optic 384-well plates [29], [30]. Samples were then processed for immunofluorescence with the indicated stains, and imaged with a 40X water immersion objective. Automated image analysis was performed as described in the text using Columbus 2.3. Texture tools such as those based on kernels of fixed size (1.25px/unit, SER parameters, PerkinElmer technologies; Haralick statistics, diameter 1.5px) were obtained, together with measurements of the regional distribution of ER staining intensity. ER staining validation was performed by correlating our chosen immunostaining with the signal obtained with a brefeldin-based probe for ER membranes and early secretory pathway structures (ER tracker, Molecular Probes; fig. S4A). Image texture values depicted in fig. 4 correspond to “SER Hole” kernel-based texture analysis.

### Viability and calcium mobilization assays

Viability assays were performed using the CellTiter Glo luminiscence assay (Promega) directly on 384-well opaque plaques (PerkinElmer), which were arrayed with the interested dsRNAs in quadruplicate, with plate replicates for each experiment. Assay was conducted as recommended by the supplier, and read on an Envision multiassay station (PerkinElmer).

Calcium mobilization assays were performed on 384-well optical plates using the Fluo-4 reagent (Molecular Probes) as recommended by the supplier. Briefly, live cells transfected for 96 h with the interested dsRNAs were stained for 30 min with a 1∶10000 dilution in fresh medium of the staining reagent in the dark, and a basal reading was obtained. Cells were then exposed to 1 µM thapsigargin for 15 min, and subsequent readings were obtained at the indicated times. Fluorescence signal was captured using an Envision multiassay station set at 485 nm excitation and 510 nm emission filter configuration.

### Estimation of total ER content

Live S2R+ cells exposed to the indicated treatments were stained for 15 min with ER tracker Blue DPX (Invitrogen) at a final concentration of 10 nM in fresh complete medium. Cells were then directly harvested, resuspended in Seecof saline buffer (6 mM Na2HPO4, 3.67 mM KH2PO4, 106 mM NaCl, 26.8 mM KCl, 6.4 mM MgCl2, 2.25 mM CaCl2, pH 6.8) plus 0.1%BSA and placed on ice for immediate analysis by flow cytometry using a LSRII station (Becton Dickinson).

### Protein analysis

Unless otherwise stated, whole cell lysates were obtained by directly exposing cells to lysis buffer (8 M Urea, 250 mM NaCl, 40 mM HEPES pH 7.9, 5% glycerol) supplemented with phosphatase and protease inhibitors (Roche). After mechanical shearing of chromatin, brief centrifugal spinning, and total protein concentration and equilibration among samples (BCA assay, ThermoScientific), a portion of sample was mixed 1∶1 with Laemmli SDS-PAGE buffer (2% SDS, 100 mM Tris HCl pH 6.8, 0.005% bromophenol blue, 2 mM DTT, 20% glycerol) and boiled for subsequent SDS-PAGE and conventional western blot analysis. Non-reducing SDS-PAGE for estimation of red/ox ratios in *Drosophila* S2R+ cells (fig. S2B) was performed by washing the cells briefly with Seecof saline buffer containing 50 mM iodoacetamide. Cells were then harvested, pelleted and directly boiled in NR buffer (2% SDS, 100 mM Tris HCl pH 6.8, 0.005% bromophenol blue, 20% glycerol, 10 mM iodoacetamide) for conventional SDS-PAGE and western blot analysis.

A protocol for fractionation of membrane-associated and nuclear subpopulations of SREBP from S2R+ cells as shown in fig. S4 was modified from [58] and references therein. Briefly, 2×10^6^ cells from each condition assayed were harvested on ice cold Seecof saline buffer, and incubated for 15 min on 200microl of hypotonic solution (20 mM HEPES-KOH, pH 8; 10 mM KCl; 1.5 mM MgCl_2_; 0.2 mM EDTA pH 8; 1 mM PMSF; 1X Protease and Phosphatase inhibitor cocktail). Plasma membrane was mechanically sheared by passing cells through a 25G needle in three strokes; integrity and purity of nuclei were assessed in an inverted light microscope. After spinning nuclei at 950 g for 15 min, the supernatant was diluted 1∶10 in hypotonic buffer and further spun at ∼100000 g in a SWTi-45 rotor for 1 h, and the pellet (membrane-bound fraction) was resuspended in 20 µl of Laemmli buffer. The nuclear pellet from the first centrifugation step was washed once in hypotonic buffer, and then extracted on ice for 30 min in 100 µl hypertonic-detergent buffer (20 mM HEPES-KOH, pH 8; 10 mM KCl; 1.5 mM MgCl_2_; 0.2 mM EDTA pH 8; 10% glycerol; 600 mM NaCl; 0.5% NP-40; 0.2 mM DTT; 1 mM PMSF; 1X Protease and Phosphatase inhibitor cocktail). After brief centrifugation at ∼20000 g, a third of the resulting cleared material was analyzed by western blot for nuclear, mature SREBP.

### Lipid analysis

After conventional organic extraction from crude cell pellets, phosphatidylcholine (PC) and phosphatidylethanolamine (PE) species were semiquantified in a Shimadzu IT-TOF mass spectrometer hyphenated with a Shimadzu Prominence HPLC system (Babraham Institute Lipidomics laboratory, UK). For the measurement of free fatty acids (FFA) release, a colorimetric reagent for measuring free glycerol levels (ZenBio, USA) was used directly on whole cell lysates from ∼2×10^5^ cells, prepared by homogeneization in HNE buffer (20 mM HEPES pH 7.9, 150 mM NaCl, 5 mM EDTA, and protease inhibitors). Colorimetric signal was measured on an Envision unit (Perkin Elmer) using a 530 nm absorbance filter. Measurements in triplicate from three independent experiments were collected for each assay, and normalized for protein content as estimated by Bradford assay.

### Statistical analysis, GO functional analysis, and HIS analysis

Data analysis, presentation and analysis of statistical significance were performed using the GraphPad Prism 4.0 software. For the quantification of XBP1 mRNA unconventional splicing by RT-PCR in the experiments shown in figures 2, 5 and 7, a minimum of two technical replicates, each including two biological replicates (hence, at least four independent runs), were analyzed for t-Student's test for the indicated sample pairs. GO functional analysis shown in figures 1 and 6 were performed with the open-source DAVID platform, using a 0.05 cut-off and establishing as background the whole query dataset. Hierarchical Interaction Score (HIS) was applied on multidimensional data from functional screenings of the XH set (Table S4). Normalized average scores for each gene were used as input using the online open-source application http://www.his2graph.net/ [47]. Graph thresholding (HIS Score≥0.13) and presentation was performed in Cytoscape 2.3.

## Supporting Information

Figure S1 **A genome-scale RNAi screen in S2R+ cells reveals regulators of ER homeostasis in normal proliferating cells.** *(A)* Schematic diagram shows how the XBP1-EGFP reporter recapitulates IRE1-dependent signaling. *(B) XBP1-EGFP* is IRE1-dependent and correlates with extent/duration of ER stress induction upon exposure to the N-glycosylation inhibitor tunicamycin. *(C)* RNAi-mediated depletion of ire1 from S2R+/XBP1-EGFP cells renders them unresponsive to genetic backgrounds that provoke alterations of ER homeostasis. Double RNAi was performed for focused screening of the XH gene set (see Table S2). Graph represents a ranking of Z-scores from averaged normalized values of two replicates. *(D)* Diagram depicts workflow for the *XBP1-EGFP* screen and details the subsets of RNAi tested. *(E)* Scatter-plot representing nuclear size [*y* axis] against Z-score [*x* axis] obtained in the wild-type screen for XBP1-EGFP regulators. Selected subsets of positive hits (G1/S control *versus* TOR signaling) are highlighted.
(TIF)

Figure S2 *(A)* qRT-PCR assessment of the knockdown efficiency of the dsRNAs targeting the indicated genes, as % reduction from wild-type levels. *(B)* Non-reducing SDS-PAGE (12.5% acrylamide) analysis correlates with observed image-based red/ox (400∶490 ratio) estimations from the eroGFP reporter in a S2R+ *Drosophila* stable cell line across different conditions (see fig. 2D–F). Graph depicts densitometry analysis averaged from two independent experiments. *NR*: non-reducing SDS-PAGE; *R*: Reducing SDS-PAGE. 10 µg from whole cell lysates were analyzed by western blotting using a commercial anti-GFP antibody. *: *p*<0.05; **: *p*<0.02; *n.s*.: non-significant.
(TIF)

Figure S3 **Inhibition of TOR-dependent signaling is associated with alterations in ER architecture and defective ER remodeling upon induction of ER stress.** *(A)* Anticalreticulin antibody exhibits significant overlap with a specific early secretory pathway staining probe. *(B)* Estimation of total ER content in S2R+ cells using ER tracker Blue DPX. Cells were either cultured in normal conditions, or exposed to Tm for 12 h; then stained for 15 min and directly analyzed by flow cytometry. *(C and D)* Additional measures of image texture from the ER channel of experiments depicted in fig. 2B and C. Haralick Variance (area diameter of 1.5 px) and “SER Edge” kernel-based feature are shown. *(E)* Flow cytometry analysis of ER content in S2R+ cells exposed for 24 h to either vehicle (DMSO), TOR inhibitory treatments (rapamycin or Torin1), or insulin (500 nM) as indicated.
(TIF)

Figure S4 **The homolog of SREBP1/2, HLH106/SREBP, is a major regulator of ER homeostasis and lipid metabolism in** ***Drosophila*** **and is activated upon acute induction of ER stress in a TORC1-dependent manner.** *(A and B)* SREBP-dependent transcriptional programs are engaged upon induction of ER stress in *Drosophila* cells, as monitored by SREBP cleavage and activation. Microarray analysis and qRTPCR of S2R+ cells challenged with Tm (1 µg/ml, 6 h) reveals up-regulation of several targets of SREBP. The right list of the heatmap details values obtained for *bona fide* targets of specific regulation during ER stress, such as XBP1 targets and RIDD targets. *(B)* Western blot analysis evidences endomembrane cleavage activation of SREBP upon exposure to ER stress. Approx. 20 µg of whole cell lysate from cells treated as indicated were resolved in 8% SDS-polyacrylamide gels and analyzed with the 3B2 monoclonal antibody (Dobrosotskaya et al., 2002). f-SREBP and m-SREBP denote full length and mature SREBP forms respectively. *(C)* qRT-PCR analysis of the levels of *bona fide* SREBP targets in three different backgrounds, during acute ER stress induction. *(D)* Western blot analysis of the cytoplasmic and nuclear fractions of S2R+ cells grown in the indicated conditions using the 3B2 monoclonal antibody, in 4–20% gradient SDS-polyacrylamide gels. f-SREBP and m-SREBP denote full length and mature SREBP forms respectively. *(E)* Viability assays assessing genetic interactions between different genes and either PEK or ATF6 branches of the UPR. Viability in each condition is expressed as % of control conditions.
(TIF)

Figure S5 **G1/S blockade is associated with defective lipid mobilization.** *(A)* Standard flow cytometry shows G1/S blockade for the indicated RNAi treatments and thymidine exposure in S2R+ cells. Relative G1, S and G2/M populations are indicated as estimated from Dean-Jett-Fox models. *(B)* ∼18 h thymidine arrest renders cells unable to mobilize lipid stores upon insulin stimulation. S2R+ cells were subjected to the indicated conditions and assessed for lipid mobilization using either conventional glycerol release [upper panel] or the image-based readout [lower panel]. t-Student's test was applied to evaluate statistical significance where indicated *: *p*<0.05; **: *p*<0.01; *n.s.*: non-significant.
(TIF)

Table S1 **dsRNA libraries used for primary** ***XBP1-EGFP*** **screens, and amplicons used in the secondary validation screens.** Z-scores from averaged normalized values, and a curated list of hits are included.
(XLSX)

Table S2 **Data from SREBP-sensitized double RNAi screens for XBP1-EGFP regulators.** Z-scores from averaged normalized values and a curated full list of hits are included.
(XLSX)

Table S3 **“XH” manually curated dsRNA set used for focused screenings included selected hits and a number of additional amplicons targeting interested genes not present in the primary screens.** Z-scores from averaged normalized values are provided for each assay, performed in duplicate.
(XLSX)

Table S4 **Data across different functional RNAi screens for the XH set, used for HIS inference and network construction (** **figure 9A** **).**
(TXT)

Table S5 **Raw relative measurements of the content in PC and PE species in wild-type S2R+ cells and SREBP-depleted cells (** **figure 5B and C** **).**
(XLSX)
