# Supplementary figures and images for: Signaling Networks Converge on TORC1-SREBP Activity to Promote Endoplasmic Reticulum Homeostasis
Source: PLoS One. 2014 Jul 9;9(7):e101164. doi: 10.1371/journal.pone.0101164 (PMC4090155; doi:10.1371/journal.pone.0101164)

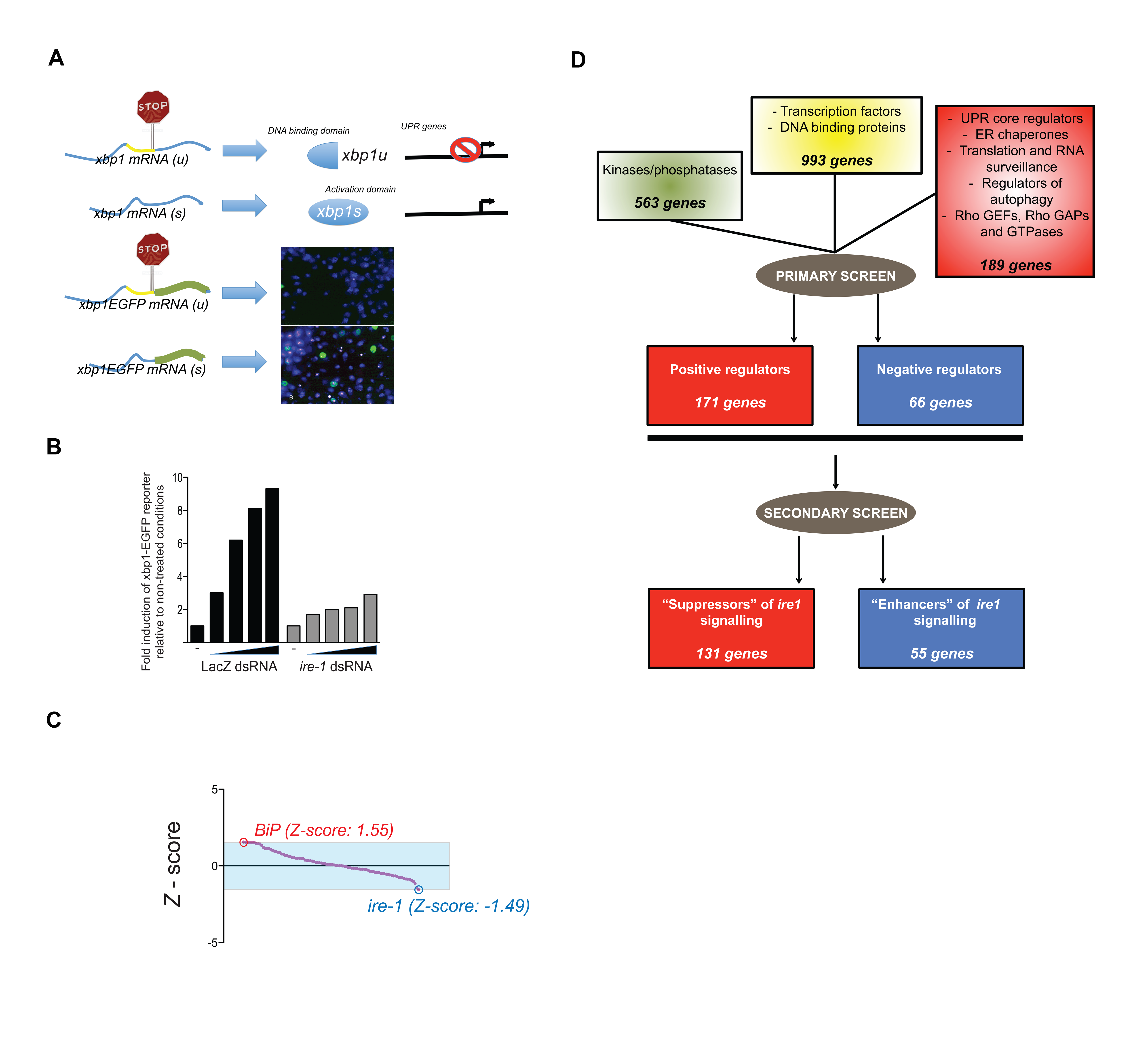

Supplement: Figure S1 — A genome-scale RNAi screen in S2R+ cells reveals regulators of ER homeostasis in normal proliferating cells. (A) Schematic diagram shows how the XBP1-EGFP reporter recapitulates IRE1-dependent signaling. (B) XBP1-EGFP is IRE1-dependent and correlates with extent/duration of ER stress induction upon exposure to the N-glycosylation inhibitor tunicamycin. (C) RNAi-mediated depletion of ire1 from S2R+/XBP1-EGFP cells renders them unresponsive to genetic backgrounds that provoke alterations of ER homeostasis. Double RNAi was performed for focused screening of the XH gene set (see Table S2). Graph represents a ranking of Z-scores from averaged normalized values of two replicates. (D) Diagram depicts workflow for the XBP1-EGFP screen and details the subsets of RNAi tested. (E) Scatter-plot representing nuclear size [y axis] against Z-score [x axis] obtained in the wild-type screen for XBP1-EGFP regulators. Selected subsets of positive hits (G1/S control versus TOR signaling) are highlighted. (TIF) [file pone.0101164.s001.tif]

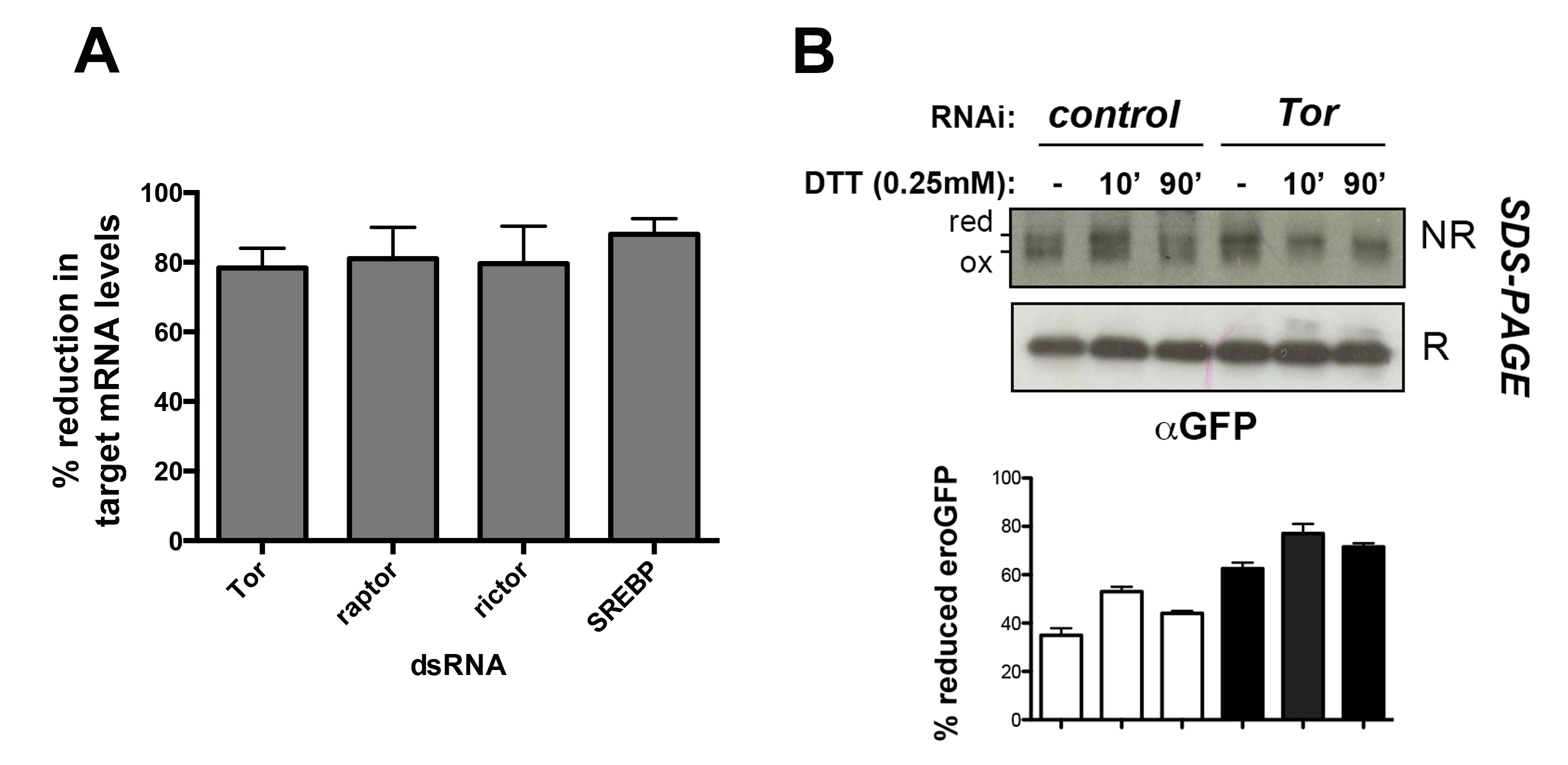

Supplement: Figure S2 — (A) qRT-PCR assessment of the knockdown efficiency of the dsRNAs targeting the indicated genes, as % reduction from wild-type levels. (B) Non-reducing SDS-PAGE (12.5% acrylamide) analysis correlates with observed image-based red/ox (400∶490 ratio) estimations from the eroGFP reporter in a S2R+ Drosophila stable cell line across different conditions (see fig. 2D–F). Graph depicts densitometry analysis averaged from two independent experiments. NR: non-reducing SDS-PAGE; R: Reducing SDS-PAGE. 10 µg from whole cell lysates were analyzed by western blotting using a commercial anti-GFP antibody. *: p<0.05; **: p<0.02; n.s.: non-significant. (TIF) [file pone.0101164.s002.tif]

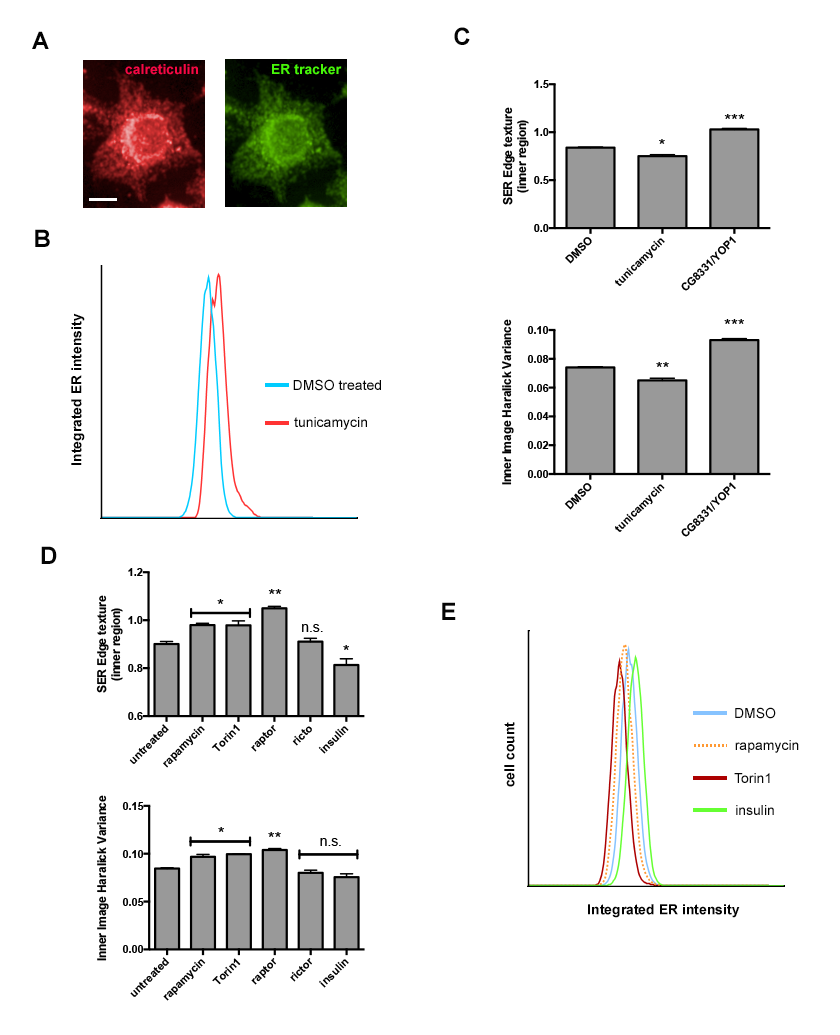

Supplement: Figure S3 — Inhibition of TOR-dependent signaling is associated with alterations in ER architecture and defective ER remodeling upon induction of ER stress. (A) Anticalreticulin antibody exhibits significant overlap with a specific early secretory pathway staining probe. (B) Estimation of total ER content in S2R+ cells using ER tracker Blue DPX. Cells were either cultured in normal conditions, or exposed to Tm for 12 h; then stained for 15 min and directly analyzed by flow cytometry. (C and D) Additional measures of image texture from the ER channel of experiments depicted in fig. 2B and C. Haralick Variance (area diameter of 1.5 px) and “SER Edge” kernel-based feature are shown. (E) Flow cytometry analysis of ER content in S2R+ cells exposed for 24 h to either vehicle (DMSO), TOR inhibitory treatments (rapamycin or Torin1), or insulin (500 nM) as indicated. (TIF) [file pone.0101164.s003.tif]

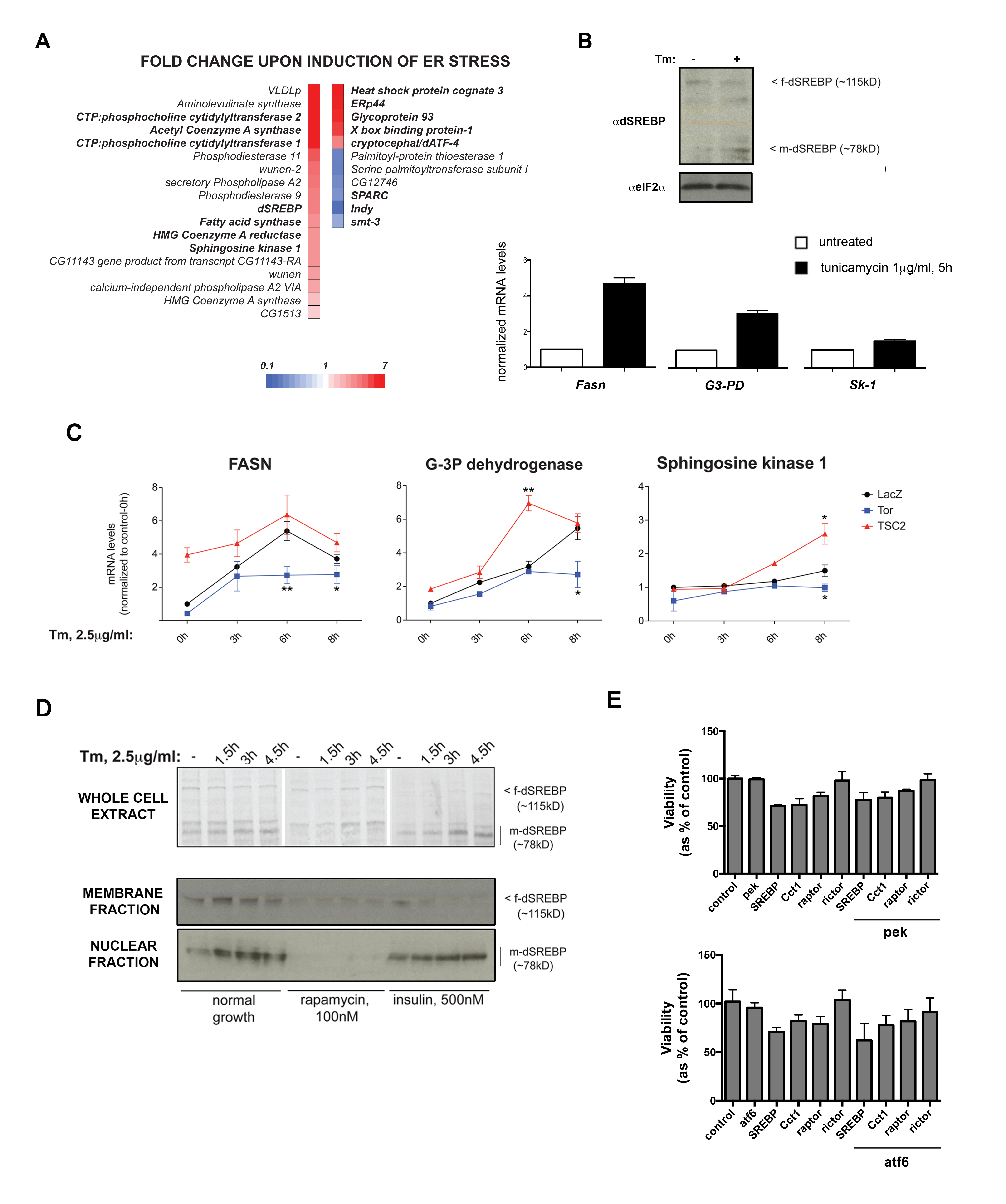

Supplement: Figure S4 — The homolog of SREBP1/2, HLH106/SREBP, is a major regulator of ER homeostasis and lipid metabolism in Drosophila and is activated upon acute induction of ER stress in a TORC1-dependent manner. (A and B) SREBP-dependent transcriptional programs are engaged upon induction of ER stress in Drosophila cells, as monitored by SREBP cleavage and activation. Microarray analysis and qRTPCR of S2R+ cells challenged with Tm (1 µg/ml, 6 h) reveals up-regulation of several targets of SREBP. The right list of the heatmap details values obtained for bona fide targets of specific regulation during ER stress, such as XBP1 targets and RIDD targets. (B) Western blot analysis evidences endomembrane cleavage activation of SREBP upon exposure to ER stress. Approx. 20 µg of whole cell lysate from cells treated as indicated were resolved in 8% SDS-polyacrylamide gels and analyzed with the 3B2 monoclonal antibody (Dobrosotskaya et al., 2002). f-SREBP and m-SREBP denote full length and mature SREBP forms respectively. (C) qRT-PCR analysis of the levels of bona fide SREBP targets in three different backgrounds, during acute ER stress induction. (D) Western blot analysis of the cytoplasmic and nuclear fractions of S2R+ cells grown in the indicated conditions using the 3B2 monoclonal antibody, in 4–20% gradient SDS-polyacrylamide gels. f-SREBP and m-SREBP denote full length and mature SREBP forms respectively. (E) Viability assays assessing genetic interactions between different genes and either PEK or ATF6 branches of the UPR. Viability in each condition is expressed as % of control conditions. (TIF) [file pone.0101164.s004.tif]

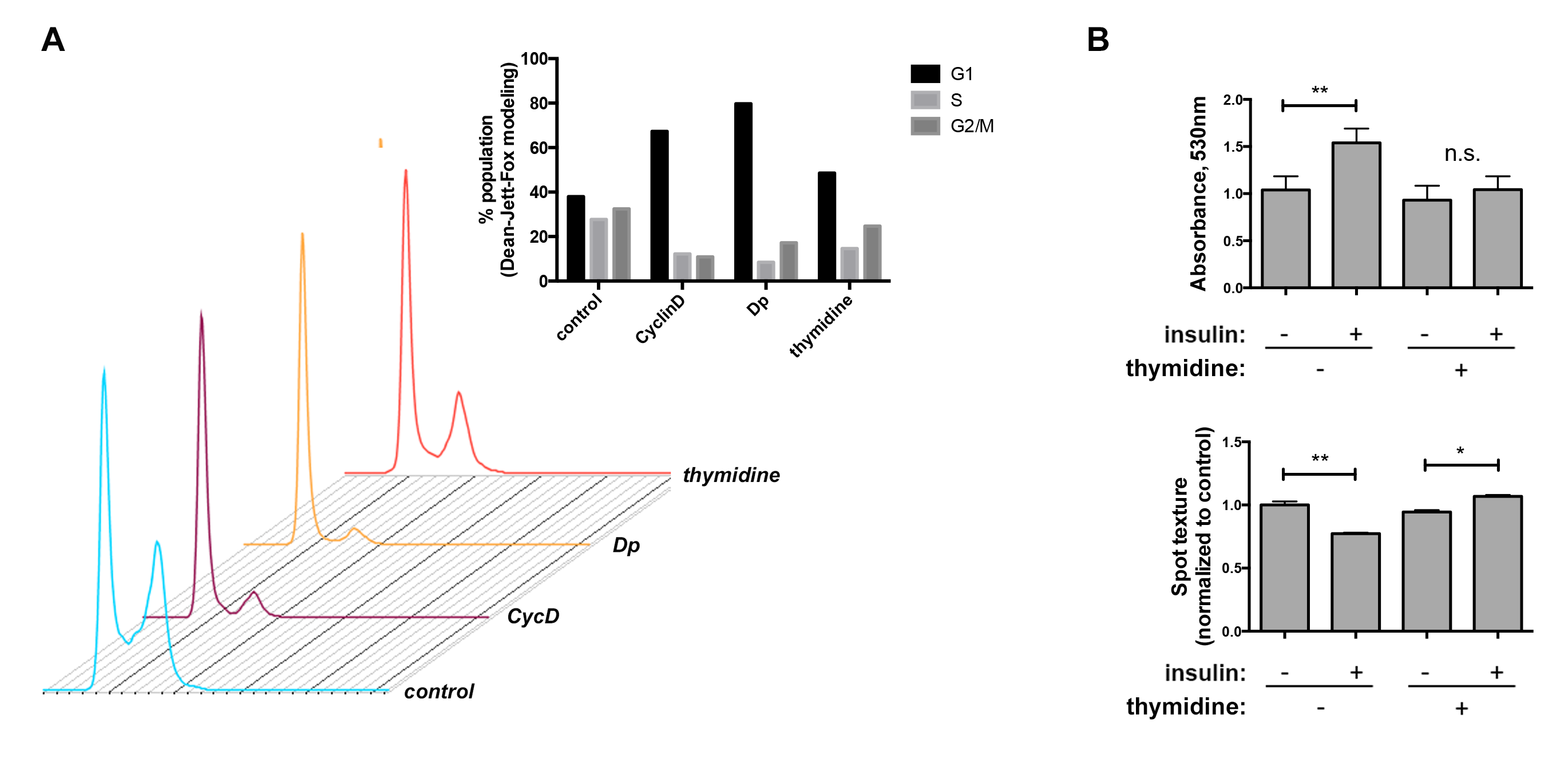

Supplement: Figure S5 — G1/S blockade is associated with defective lipid mobilization. (A) Standard flow cytometry shows G1/S blockade for the indicated RNAi treatments and thymidine exposure in S2R+ cells. Relative G1, S and G2/M populations are indicated as estimated from Dean-Jett-Fox models. (B) ∼18 h thymidine arrest renders cells unable to mobilize lipid stores upon insulin stimulation. S2R+ cells were subjected to the indicated conditions and assessed for lipid mobilization using either conventional glycerol release [upper panel] or the image-based readout [lower panel]. t-Student's test was applied to evaluate statistical significance where indicated *: p<0.05; **: p<0.01; n.s.: non-significant. (TIF) [file pone.0101164.s005.tif]
